# Correction: Global extent and change in human modification of terrestrial ecosystems from 1990 to 2022

**DOI:** 10.1038/s41597-025-05710-5

**Published:** 2025-08-07

**Authors:** David M. Theobald, James R. Oakleaf, Glenn Moncrieff, Maria Voigt, Joe Kiesecker, Christina M. Kennedy

**Affiliations:** 1 Conservation Planning Technologies, Fort Collins, CO 80521 USA; 2 Dept. of Fish, Wildlife and Conservation Biology, Colorado State University, Fort Collins, CO 80526 USA; 3 Global Protect Oceans, Lands and Waters, The Nature Conservancy, Fort Collins, CO 80524 USA; 4 Global Science, The Nature Conservancy, Cape Town, South Africa; 5 Global Science, The Nature Conservancy, Berlin, Germany; 6 Global Science, The Nature Conservancy, Fort Collins, CO 80521 USA

Correction to: *Scientific Data* 10.1038/s41597-025-04892-2, published online 10 April 2025

In the version of the article initially published, the image shown as Fig. 2 was an inadvertent duplicate of Fig. 3. Fig 2. has now been corrected in the HTML and PDF versions of the article.

