# Supplementary material for: Global extent and change in human modification of terrestrial ecosystems from 1990 to 2022
Source: Sci Data. 2025 Apr 10;12:606. doi: 10.1038/s41597-025-04892-2 (PMC11985953; doi:10.1038/s41597-025-04892-2)
Supplement: Supplementary file 1 — Supplementary information [file 41597_2025_4892_MOESM1_ESM.pdf]

Supplementary information on the global extent and change in human modification of terrestrial ecosystems from 1990 to 2022

David M. Theobald[1,2], dmt@davidmtheobald.com
James R. Oakleaf[3], joakleaf@tnc.org
Glenn Moncrieff[4], glenn.moncrieff@tnc.org
Maria Voigt[5], maria.voigt@tnc.org
Joe Kiesecker[3], jkiesecker@tnc.org
Christina M. Kennedy[6], ckennedy@tnc.org

[1]Conservation Planning Technologies, Fort Collins, CO, 80521, USA
[2] Dept. of Fish, Wildlife and Conservation Biology, Colorado State University, Fort Collins, CO, 80526, USA
[3] Global Protect Oceans, Lands and Waters, The Nature Conservancy, Fort Collins, CO, 80524, USA
[4] Global Science, The Nature Conservancy, Cape Town, South Africa
[5] Global Science, The Nature Conservancy, Berlin, Germany
[6] Global Science, The Nature Conservancy, Fort Collins, CO, 80521, USA

Supplementary Figure 1. Global human modification for 2022, with example cities: A=Asuncion, Paraguay, B=Calgary, Canada; C=Qing Yuan, China; and D: Timbuktu, Mali. Note: datasets can be explored at: https://hm-30x30.projects.earthengine.app/view/hm-v3.)

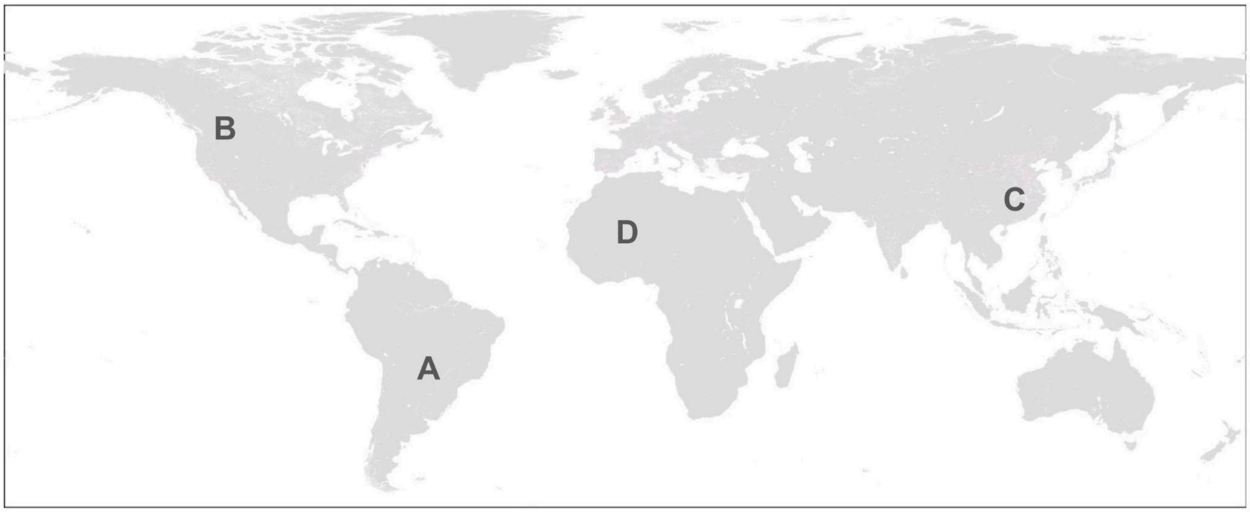

Supplementary Figure 2. Maps of human modification at 300 m for four example landscapes and their associated H for 2022 within 100 km radius of: (a, b) Asuncion, Paraguay; (c, d) Calgary, Canada; (e, f) Qing Yuan, China; and (g, h) Timbuktu, Mali. (Note: datasets can be visualized at: https://hm-30x30.projects.earthengine.app/view/hm-v3.

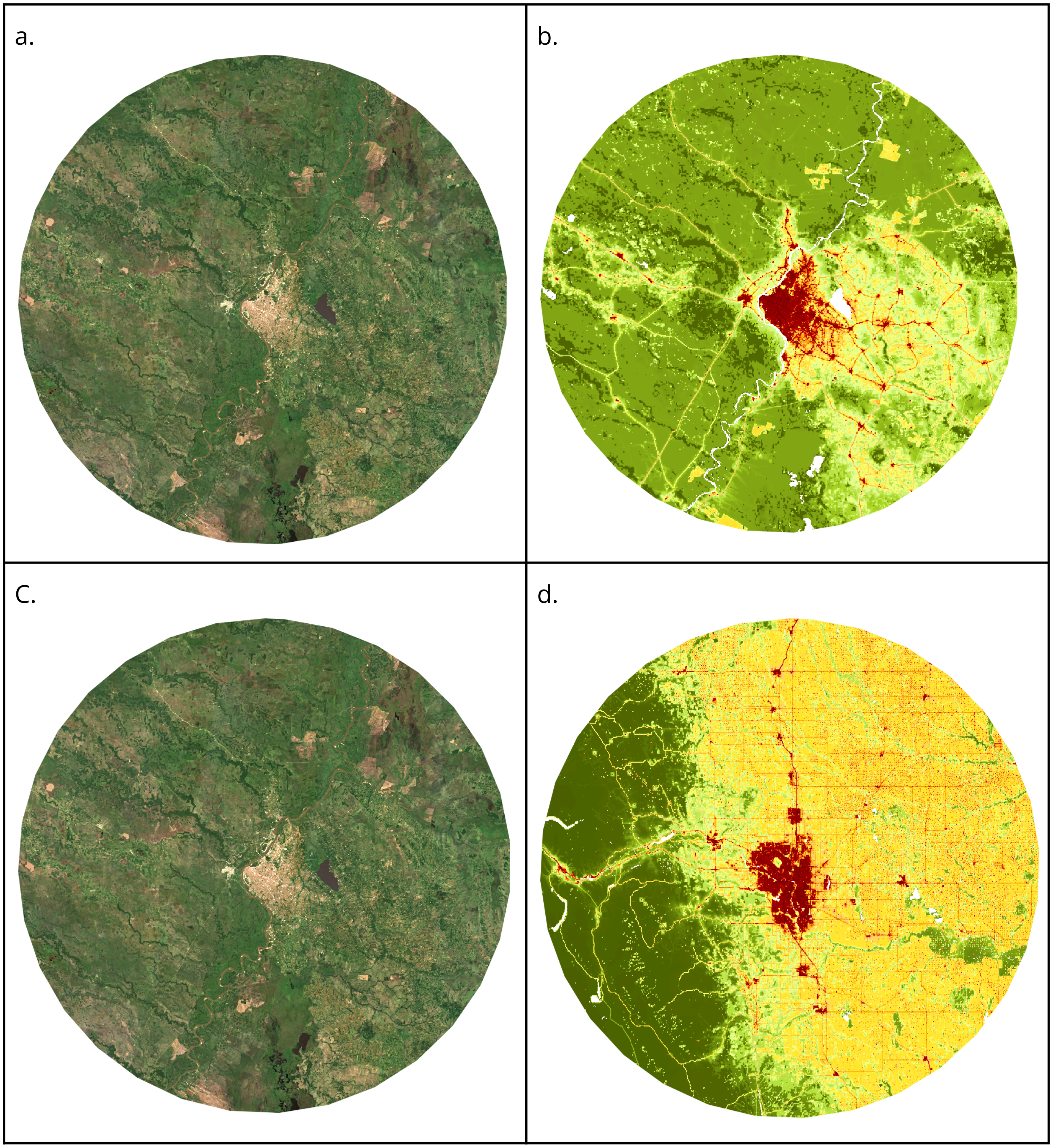

e.

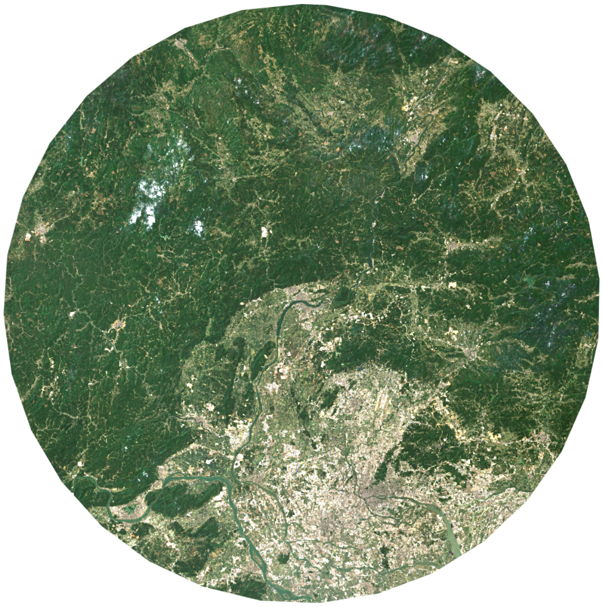

f.

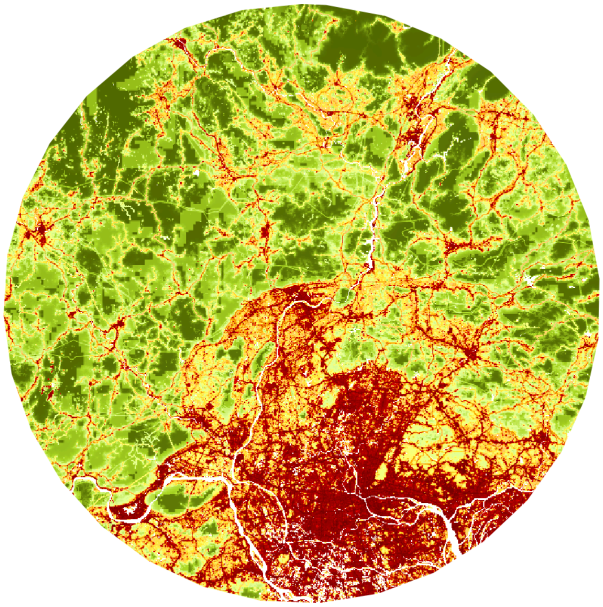

g.

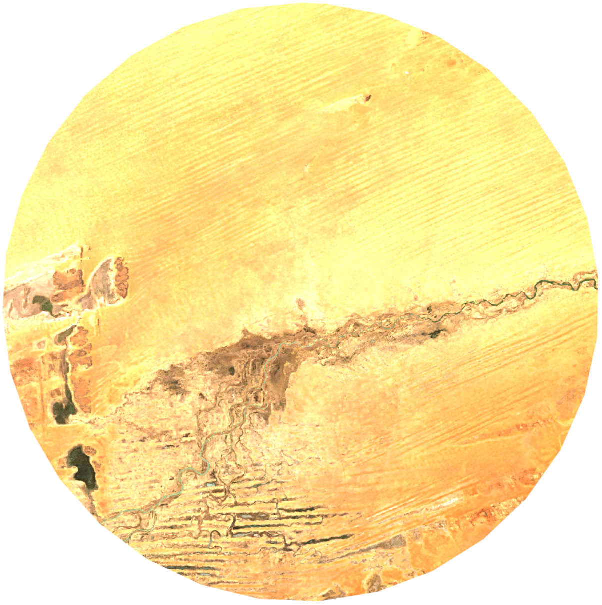

h.

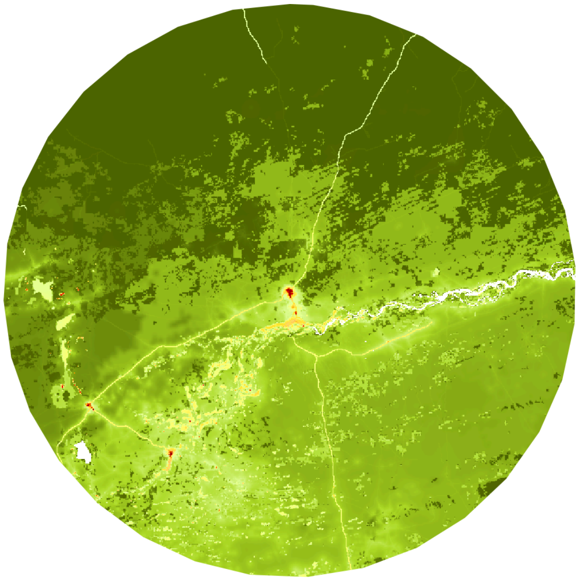

Supplementary Figure 3. Maps of human modification at four example landscapes and their associated H for 2022 within 100 km radius: (a) Asuncion, Paraguay at 300 m & (b) Asuncion, Paraguay at 90 m; (c & d) Calgary, Canada; (c) Qing yuan, China; and Timbuktu, Mali. (Note: datasets can be visualized at: https://hm-30x30.projects.earthengine.app/view/hm-v3.

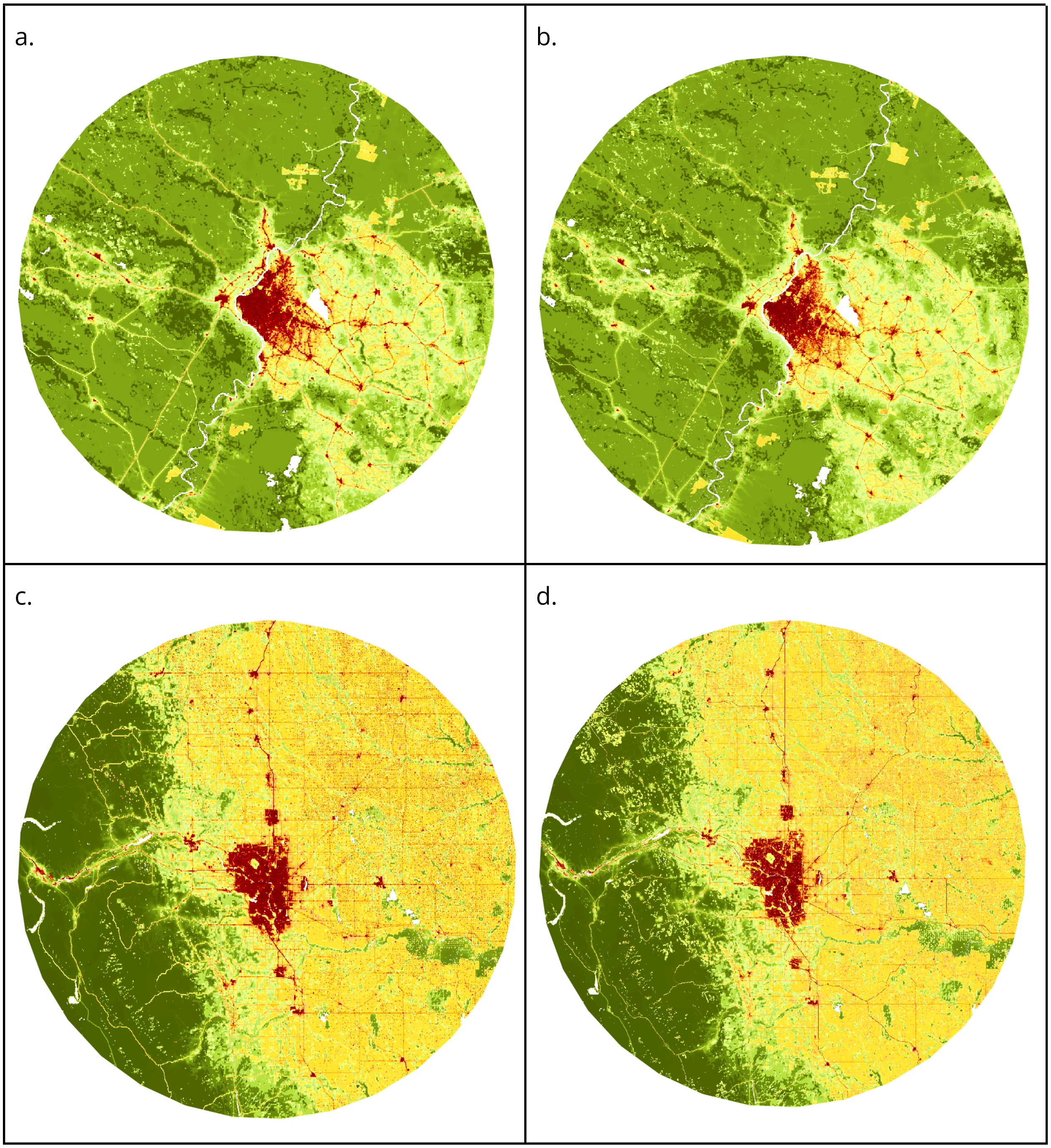

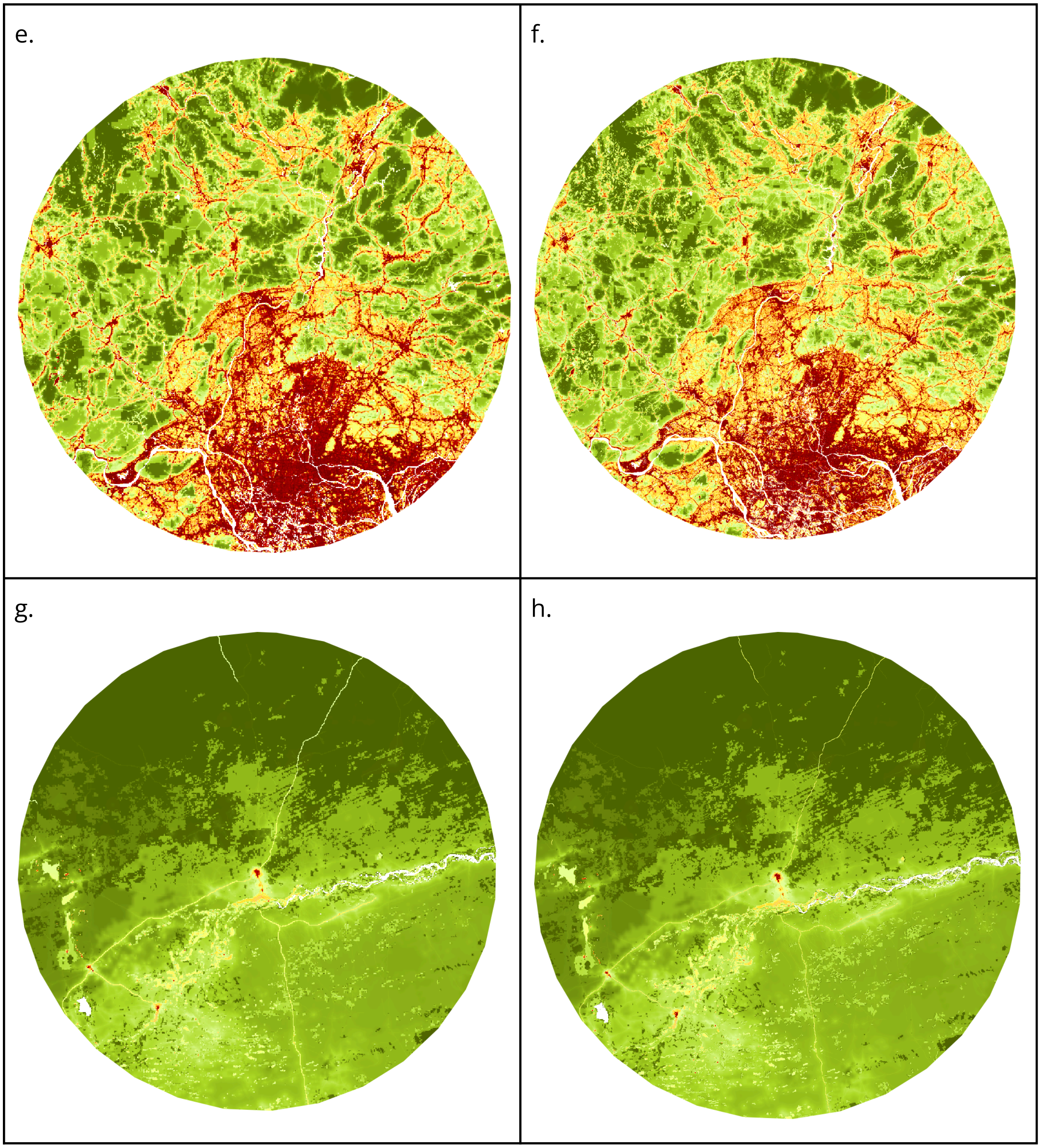
e.
f.
g.
h.

Supplementary Figure 4. Maps of human modification within 100 km radius of Asuncion, Paraguay for 1990, 2000, 2010, and 2020 (Note: datasets can be visualized at: https://hm-30x30.projects.earthengine.app/view/hm-v3.

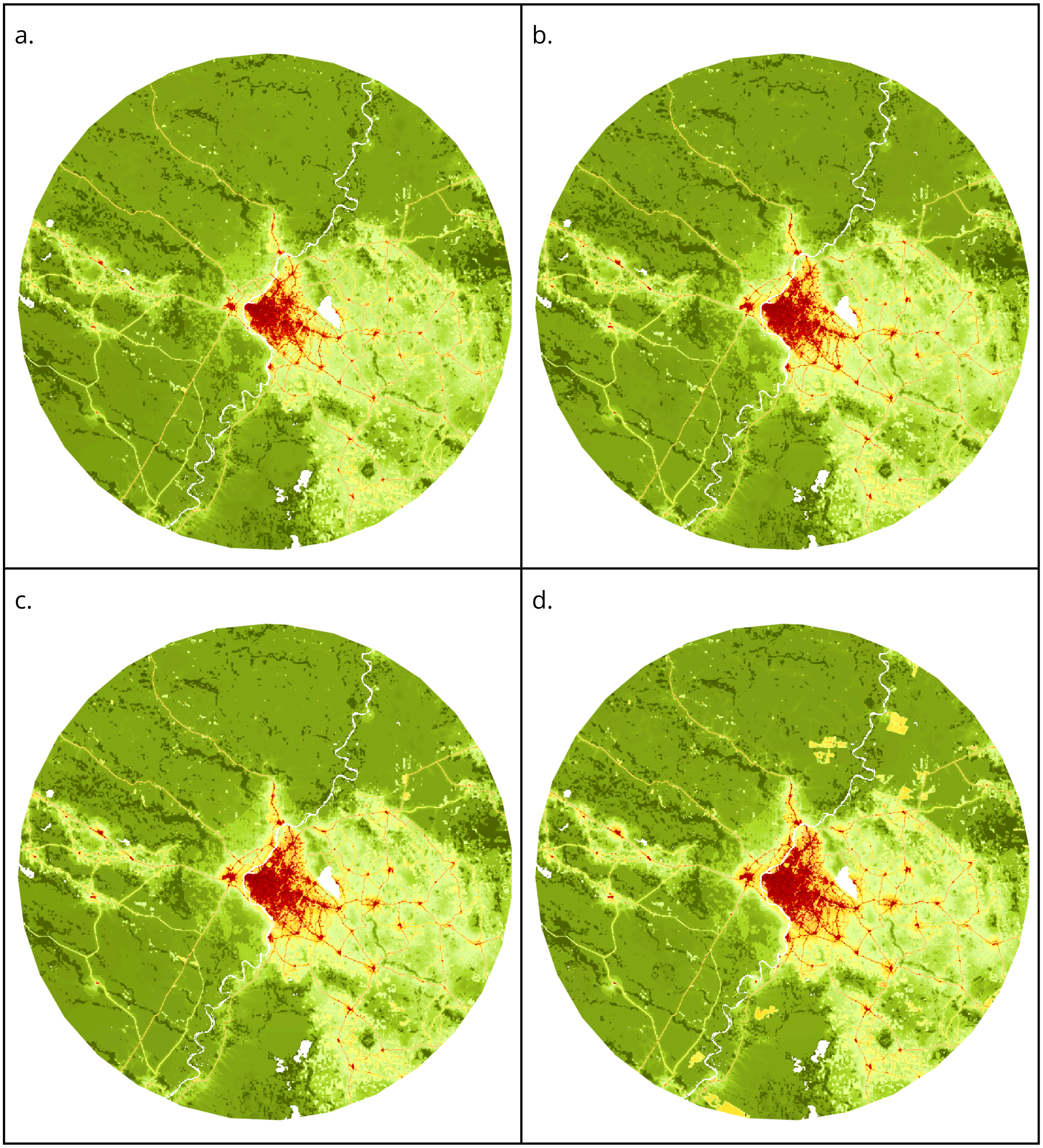

Supplementary Figure 5. Maps of human modification within 100 km radius of Calgary, Canada for 1990, 2000, 2010, and 2020. (Note: datasets can be visualized at: https://hm-30x30.projects.earthengine.app/view/hm-v3.

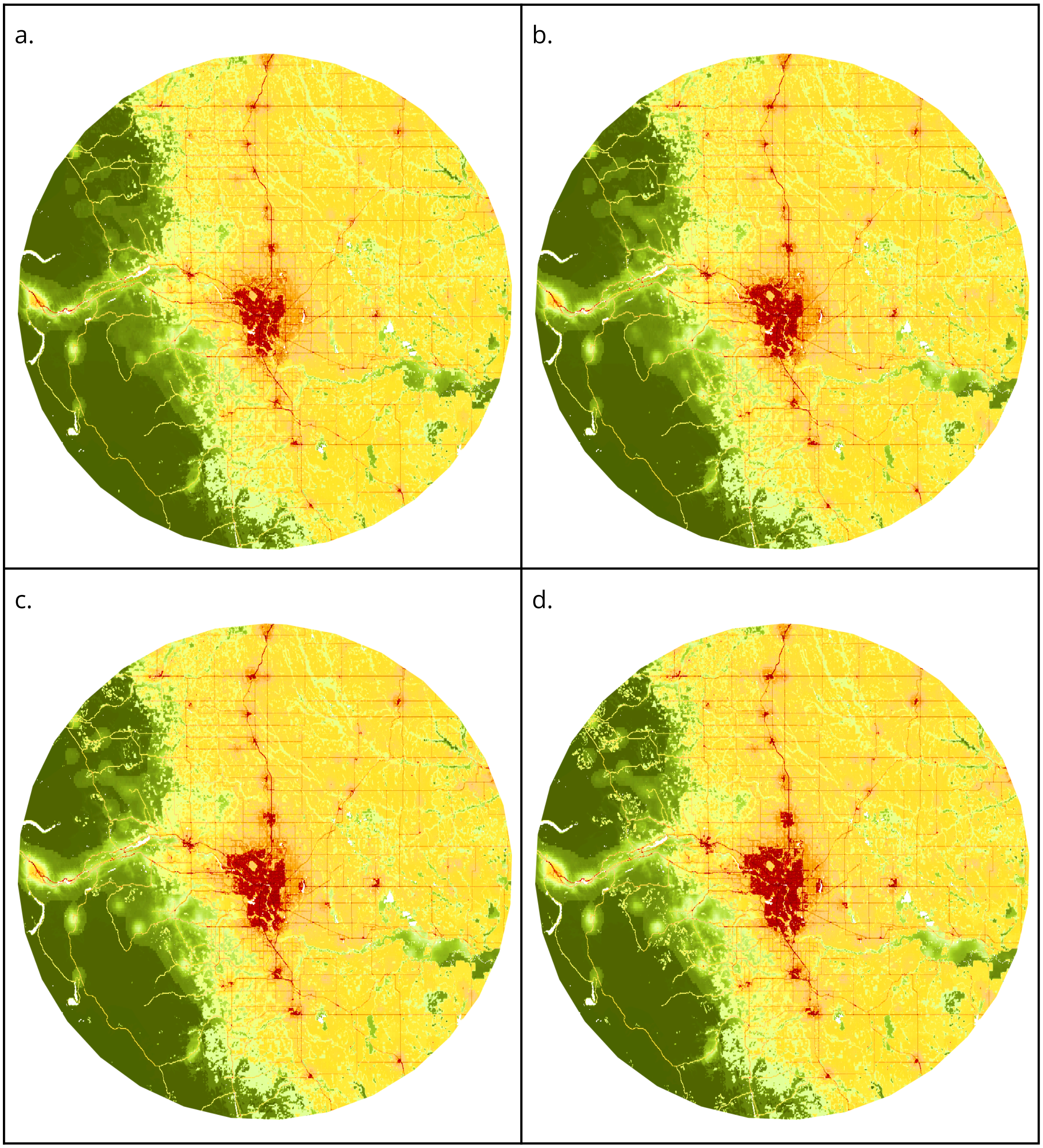

Supplementary Figure 6. Maps of human modification within 100 km radius of Qing Yuan, China for 1990, 2000, 2010, and 2020. Note: datasets can be visualized at: https://hm-30x30.projects.earthengine.app/view/hm-v3.

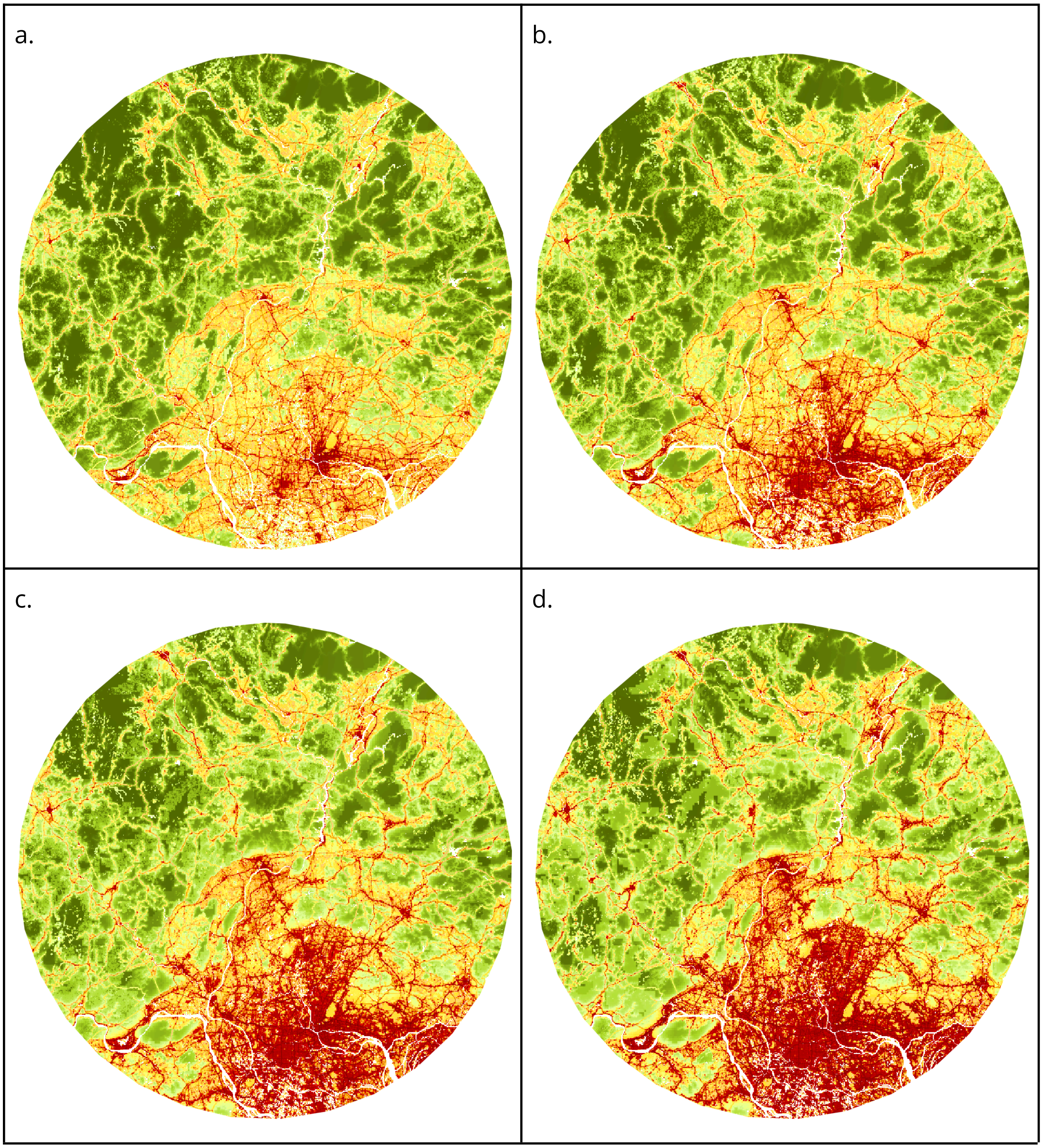

Supplementary Figure 7. Maps of human modification within 100 km radius of Timbuktu, Mali for 1990, 2000, 2010, and 2020. Note: datasets can be visualized at: https://hm-30x30.projects.earthengine.app/view/hm-v3.

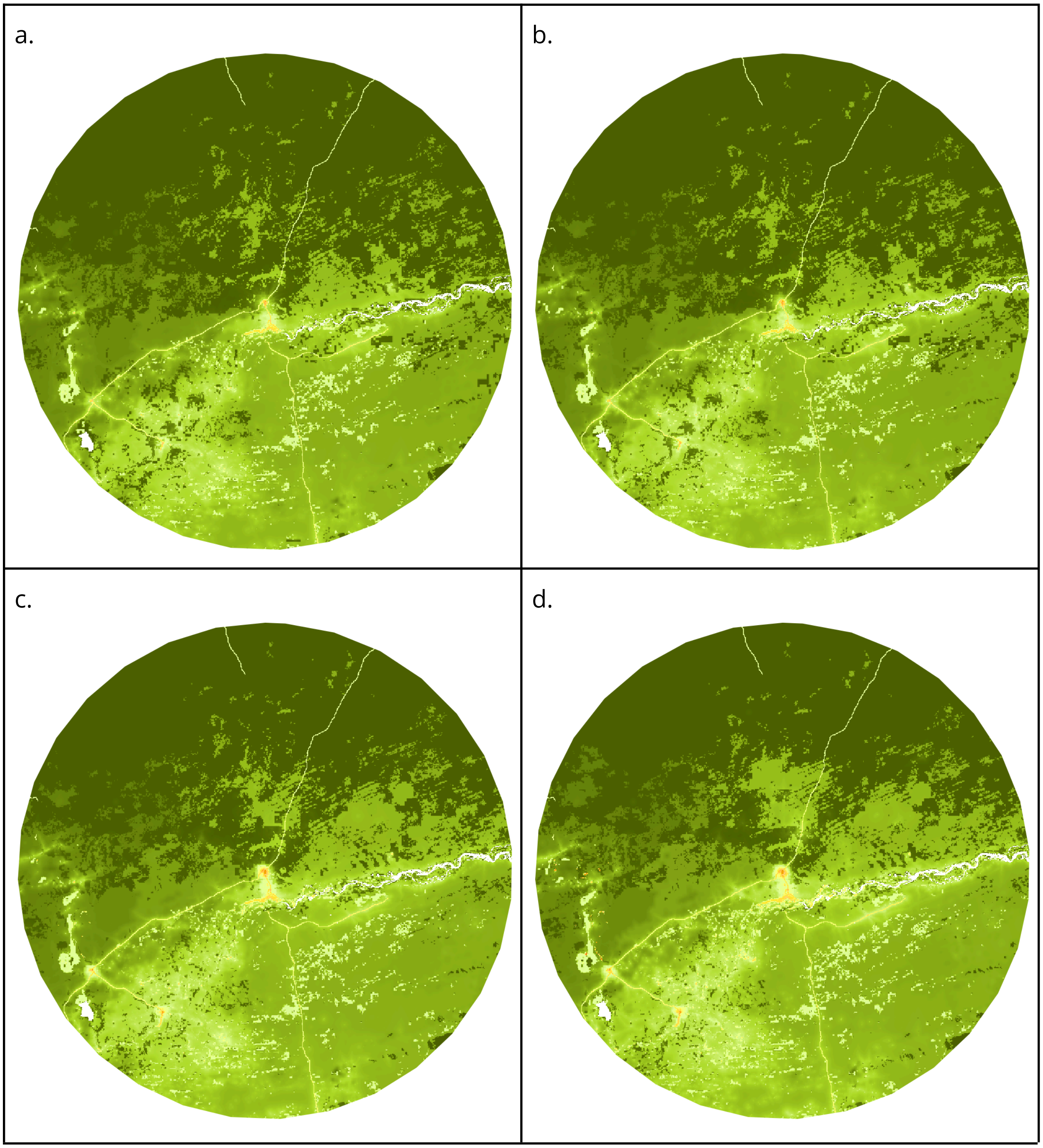

Supplementary Figure 8. Comparison of human modification and related datasets within 100 km radius of Asuncion, Paraguay for: (a) reference image from Sentinel 2; (b) HMv3 (300 m); (c) HM v2 (Theobald et al. 2020, 300 m); (d) HM v1 (Kennedy et al. 2024, 1 km); (e) HF for 2009 (Venter et al. 2016, 1 km); (f) HF for 2013 (Williams et al. 2020, 300 km); (g) HF for 2020 (Mu et al. 2022, 100 m), and (h) HF 100 m (Gassert et al. 2022). Note: datasets can be visualized globally at: https://hm-30x30.projects.earthengine.app/view/hm-v3.

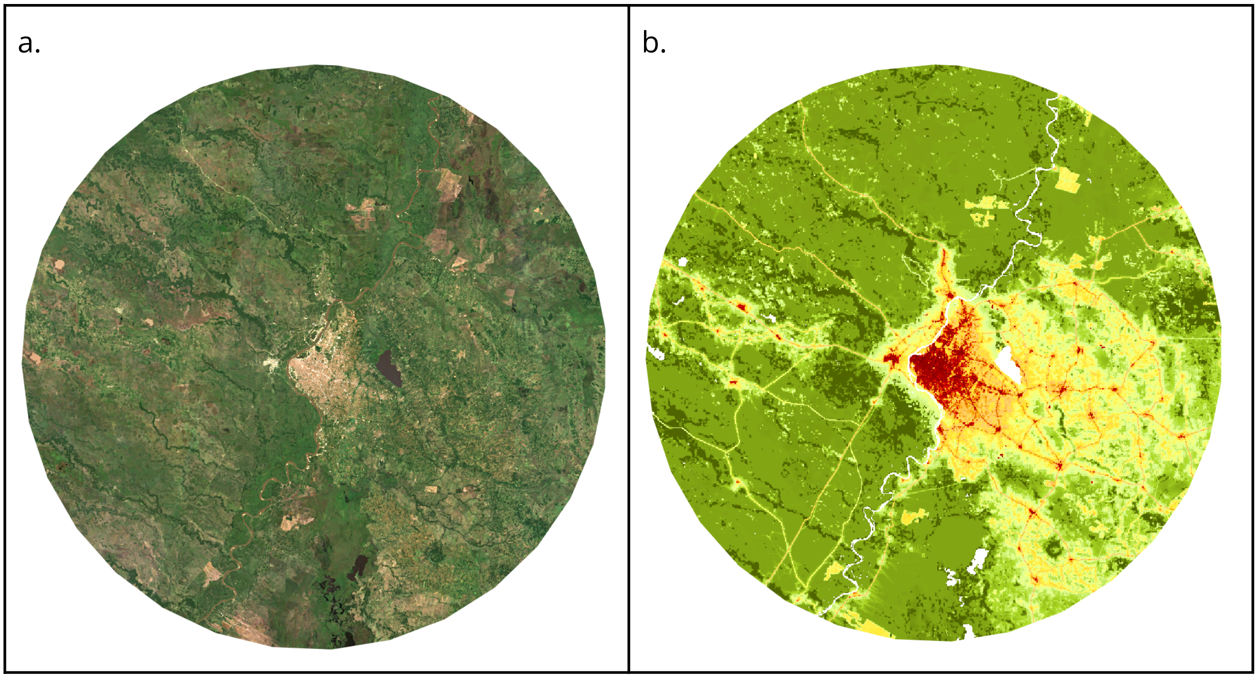

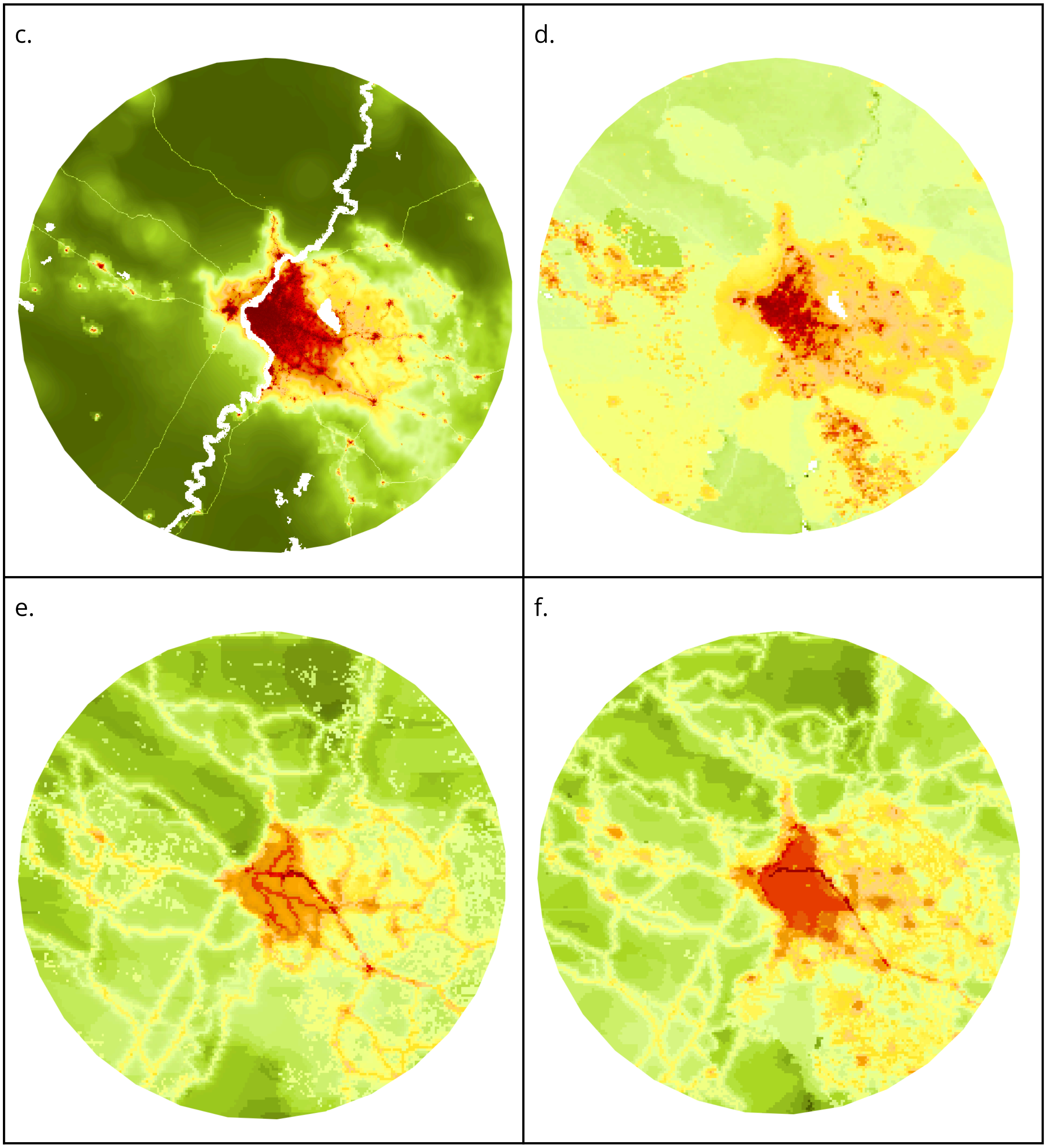
c.
d.
e.
f.

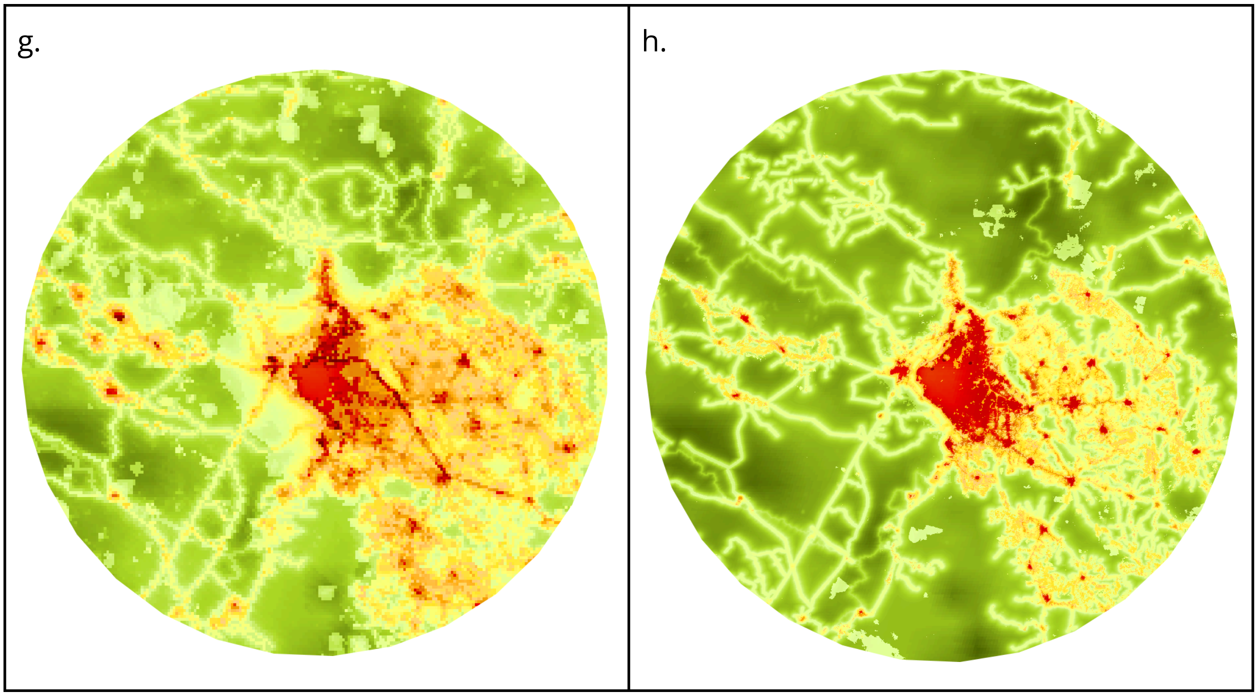
g.
h.

Supplementary Figure 9. Comparison of human modification and related datasets within 100 km radius of Calgary, Canada for: (a) reference image from Sentinel 2; (b) HMv3 (300 m); (c) HM v2 (Theobald et al. 2020, 300 m); (d) HM v1 (Kennedy et al. 2024, 1 km); (e) HF for 2009 (Venter et al. 2016, 1 km); (f) HF for 2013 (Williams et al. 2020, 300 km); (g) HF for 2020 (Mu et al. 2022, 100 m), and (h) HF 100 m (Gassert et al. 2022). Note: datasets can be visualized globally at: https://hm-30x30.projects.earthengine.app/view/hm-v3.

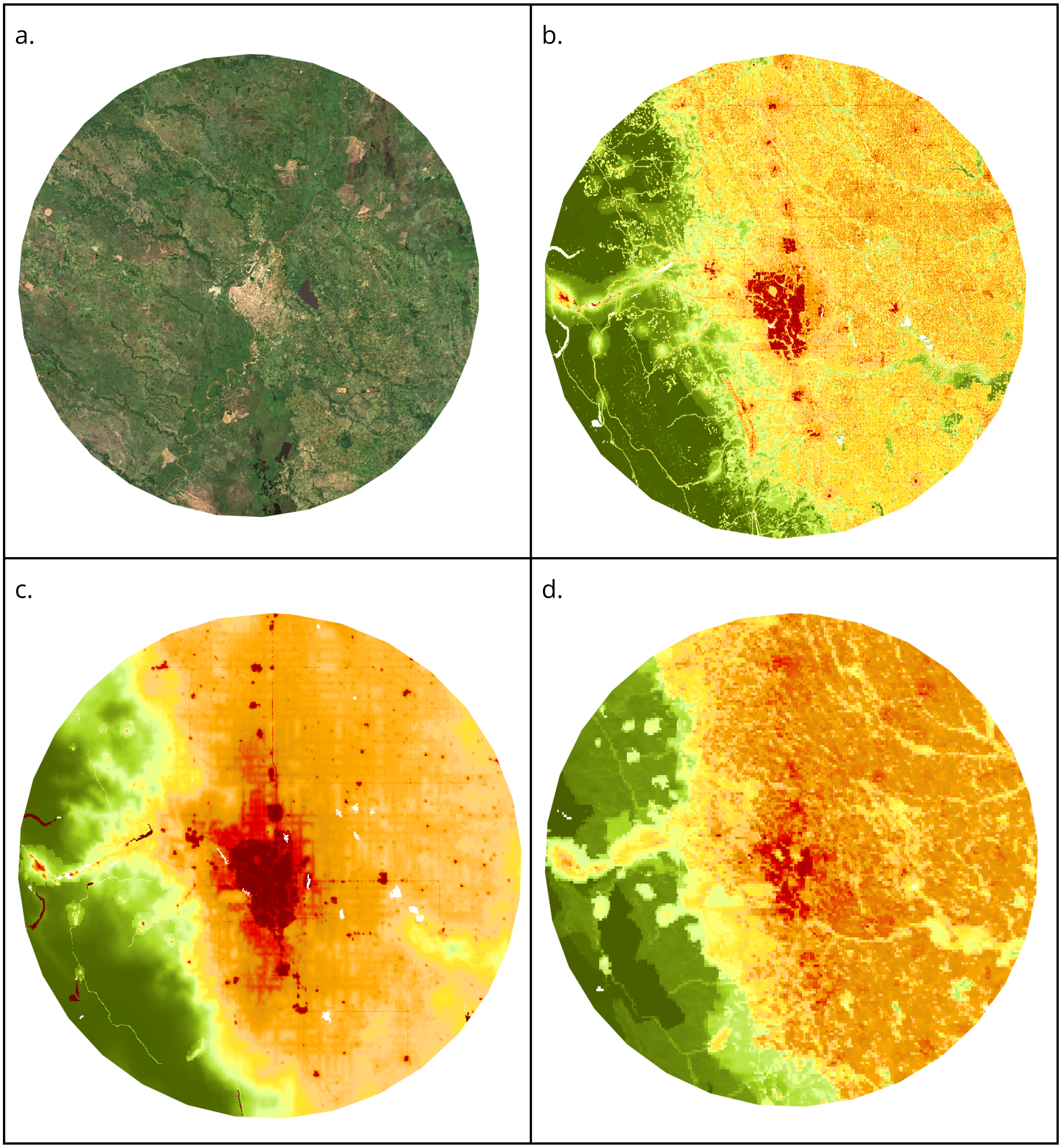

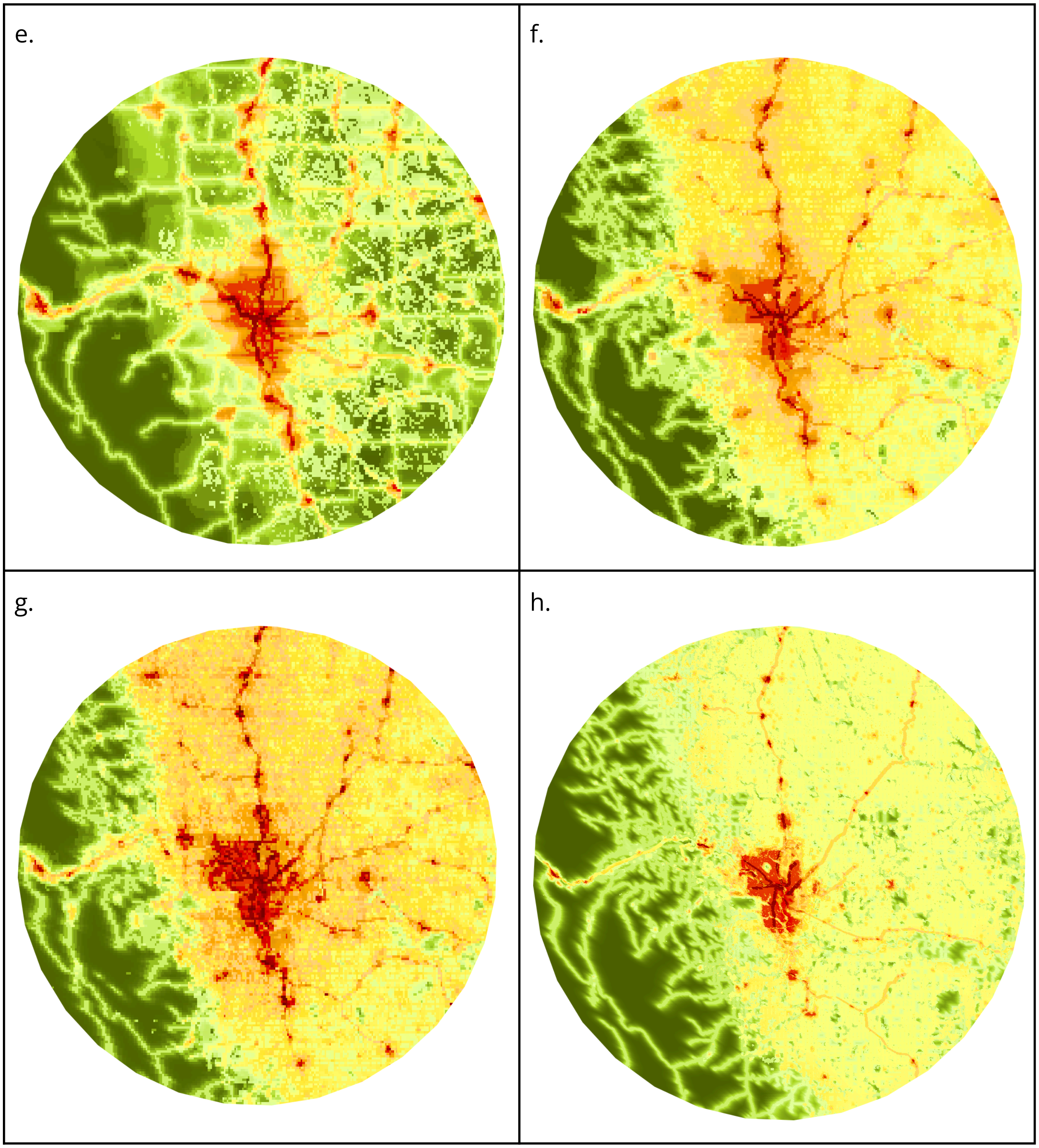
e.
f.
g.
h.

Supplementary Figure 10. Comparison of human modification and related datasets within 100 km radius of Qing Yuan, China for: (a) reference image from Sentinel 2; (b) HMv3 (300 m); (c) HM v2 (Theobald et al. 2020, 300 m); (d) HM v1 (Kennedy et al. 2024, 1 km); (e) HF for 2009 (Venter et al. 2016, 1 km); (f) HF for 2013 (Williams et al. 2020, 300 km); (g) HF for 2020 (Mu et al. 2022, 100 m), and (h) HF 100 m (Gassert et al. 2022). Note: datasets can be visualized globally at: https://hm-30x30.projects.earthengine.app/view/hm-v3.

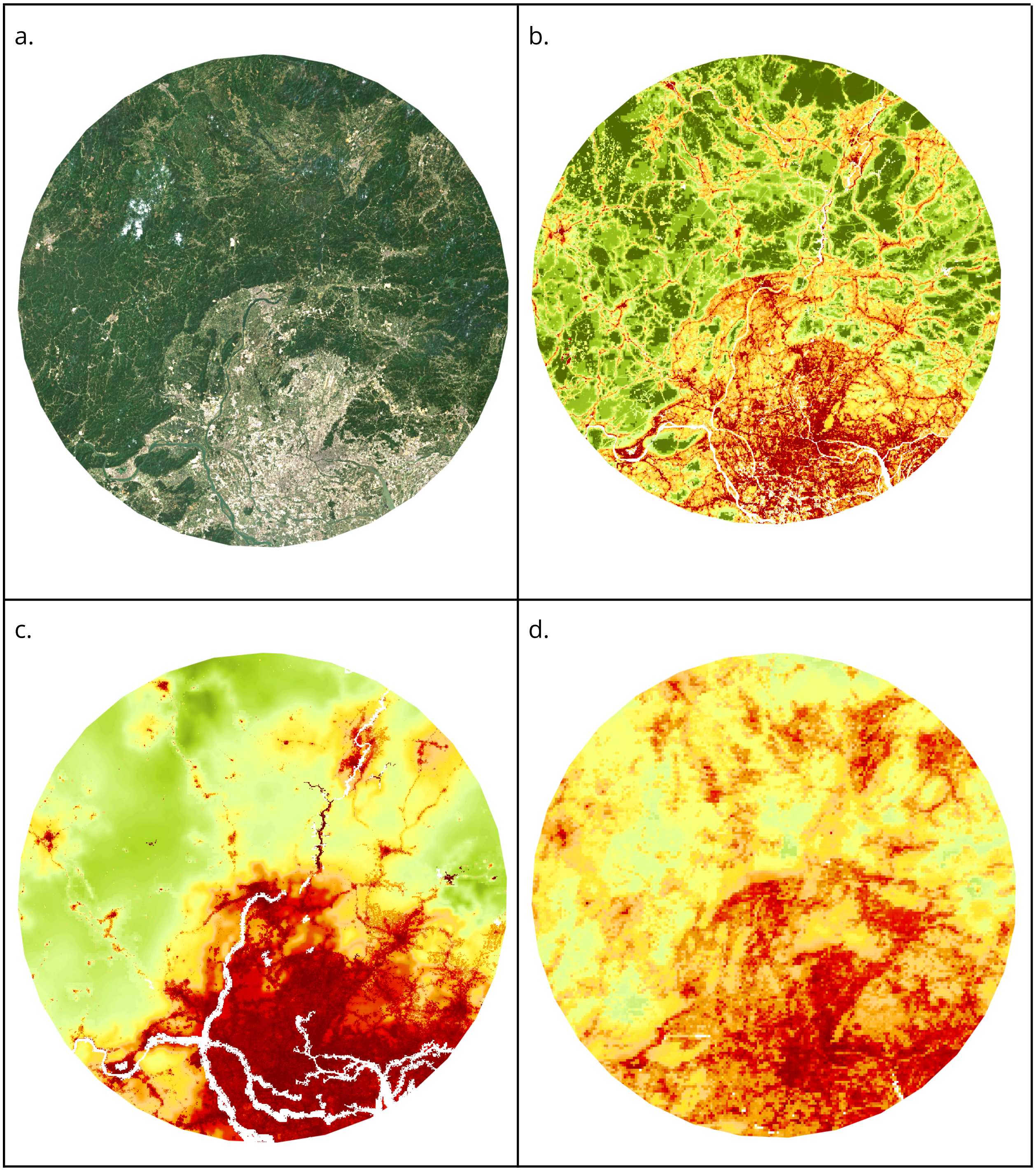

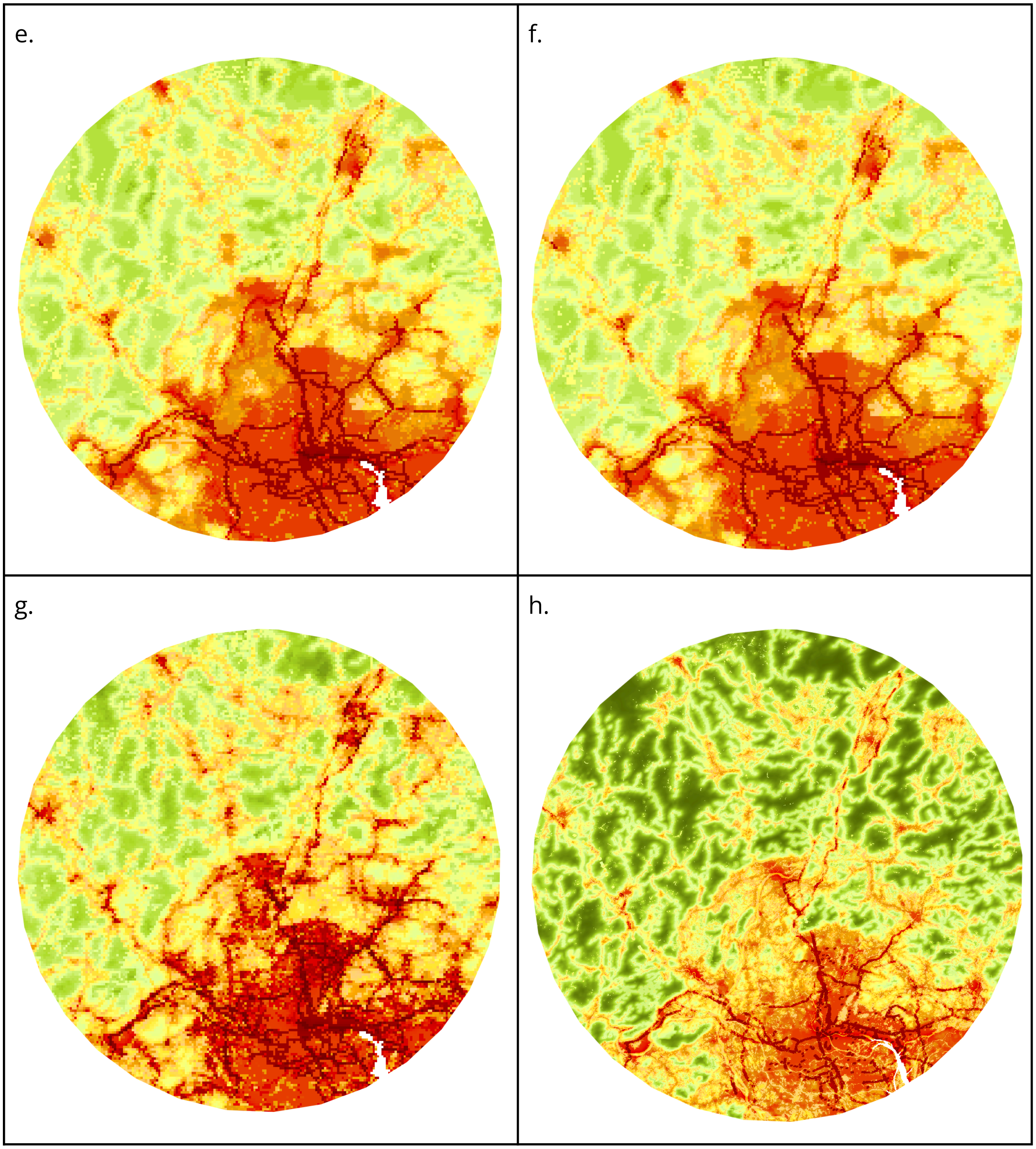
e.
f.
g.
h.

Supplementary Figure 11. Comparison of human modification and related datasets within 100 km radius of Timbuktu, Mali for: (a) reference image from Sentinel 2; (b) HMv3 (300 m); (c) HM v2 (Theobald et al. 2020, 300 m); (d) HM v1 (Kennedy et al. 2024, 1 km); (e) HF for 2009 (Venter et al. 2016, 1 km); (f) HF for 2013 (Williams et al. 2020, 300 km); (g) HF for 2020 (Mu et al. 2022, 100 m), and (h) HF 100 m (Gassert et al. 2022). Note: datasets can be visualized globally at: https://hm-30x30.projects.earthengine.app/view/hm-v3.

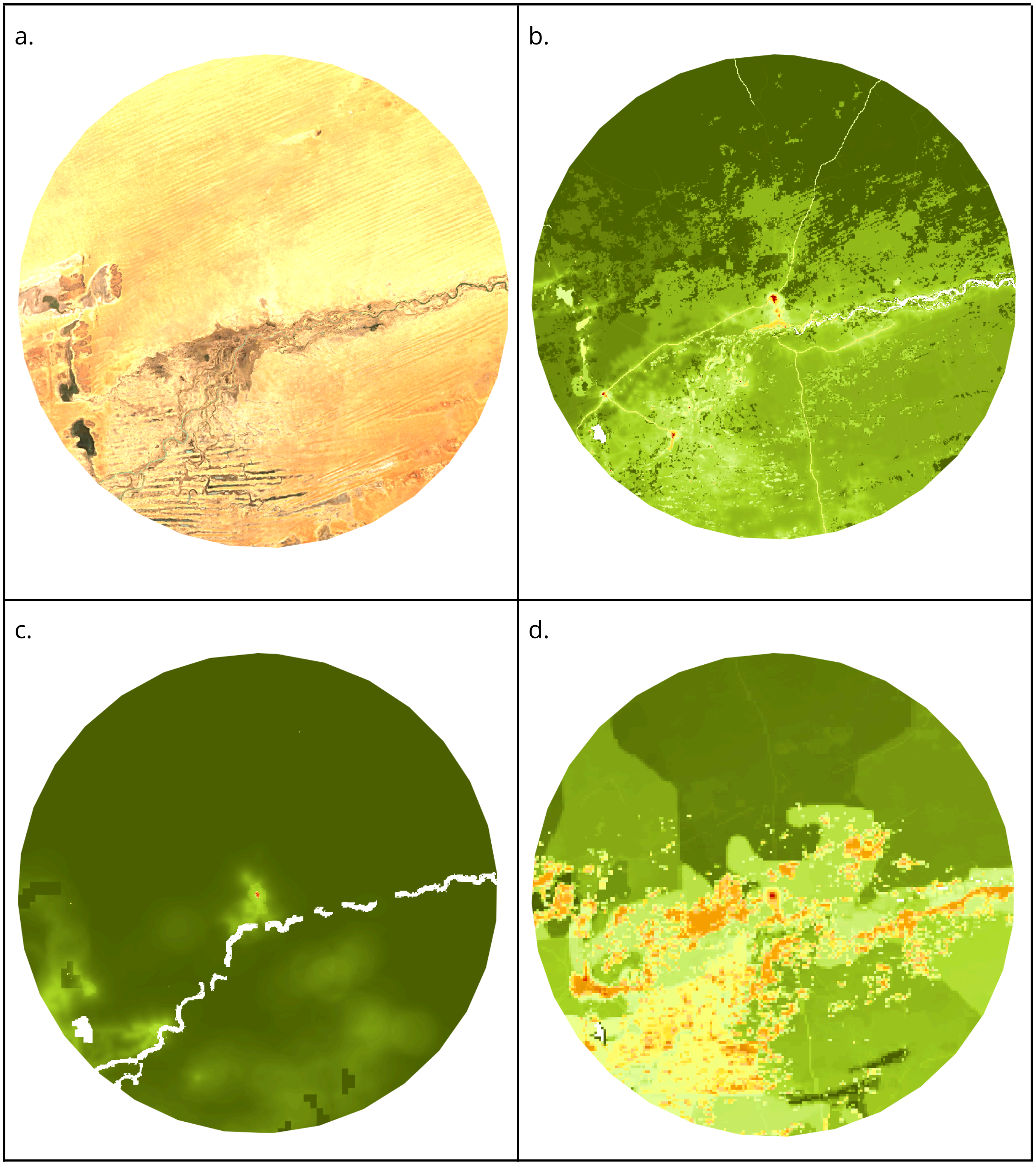

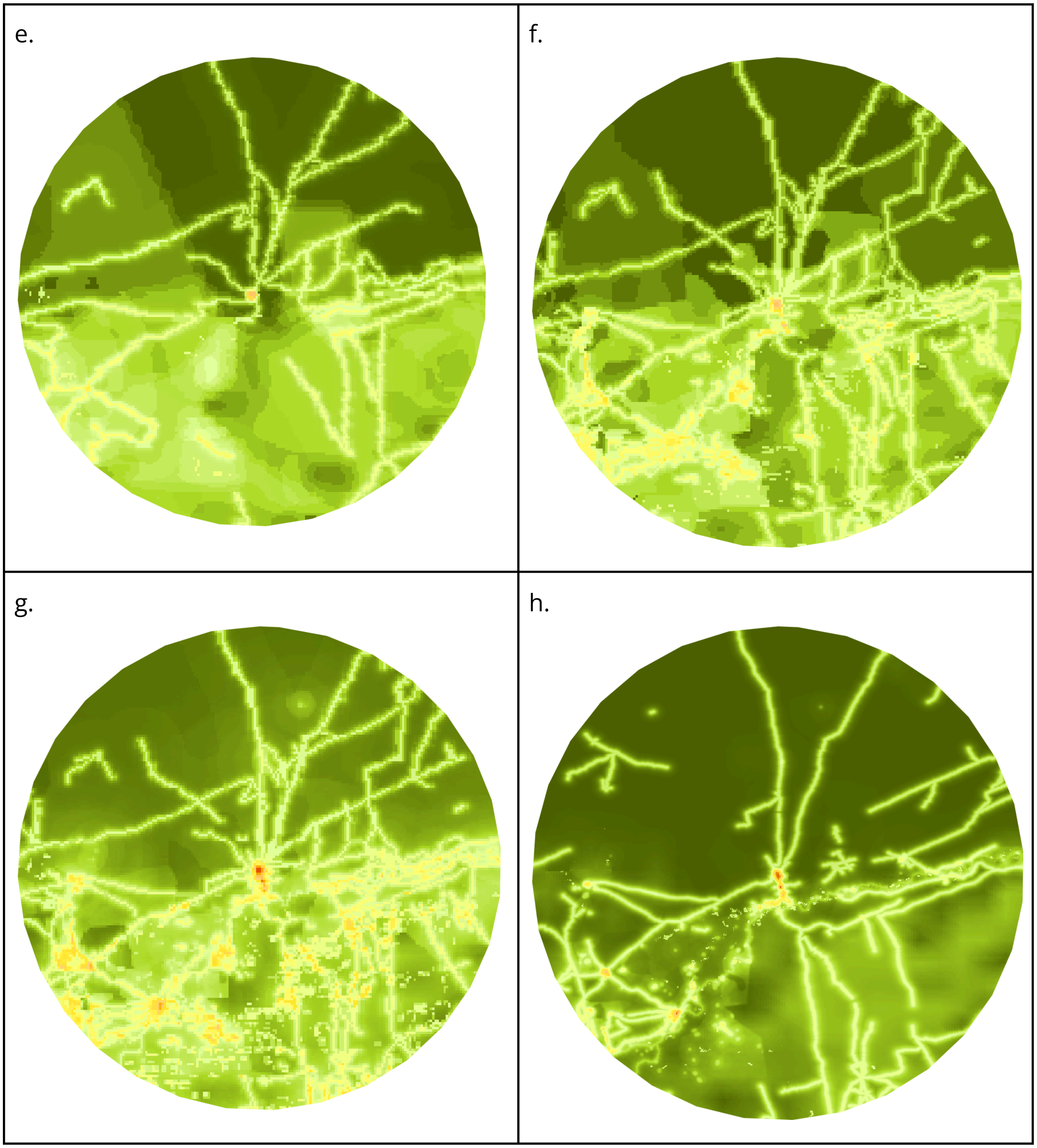
e.
f.
g.
h.

Supplementary Figure 12. Map showing which of the threat classes was “dominant” (i.e. had the highest *H* value, that exceeded 0.01), globally (top) and for the central part of North America.

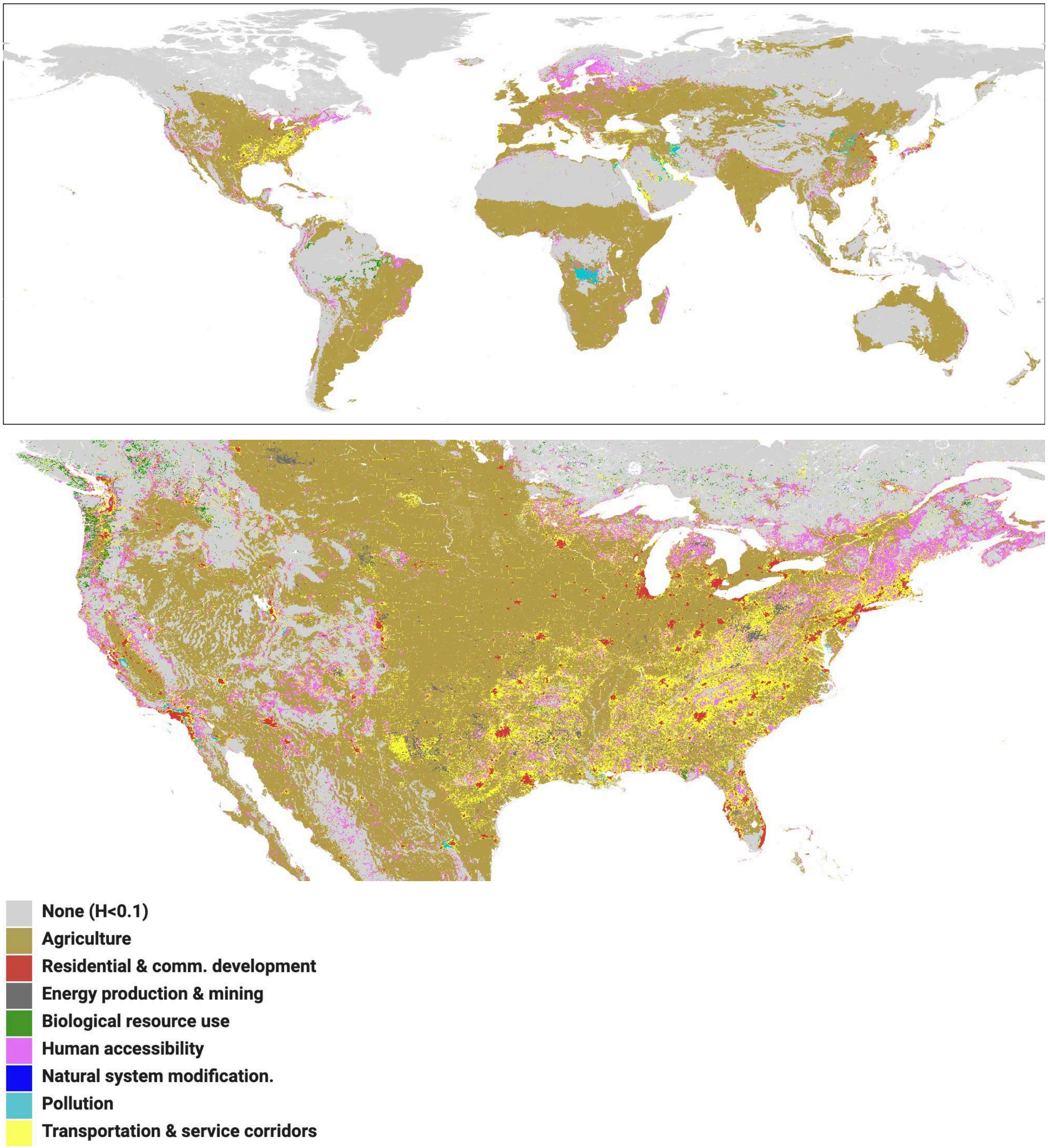

Supplementary Figure 13. Scattergram of countries depicting the percent protected vs. the increase in human modification from 1990-2020.

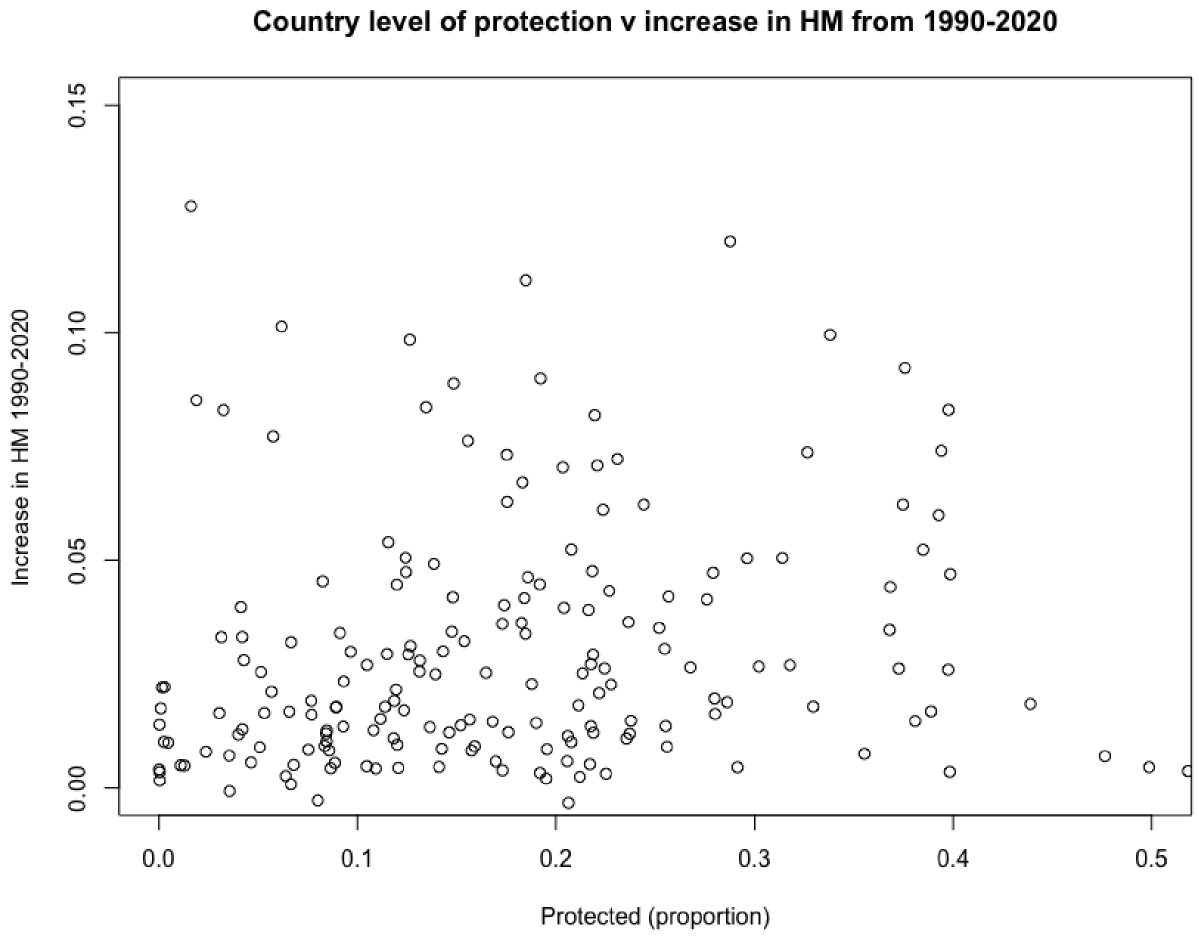

Supplementary Figure 14. Scattergram of ecoregions depicting the proportion protected vs. the increase in human modification from 1990-2020.

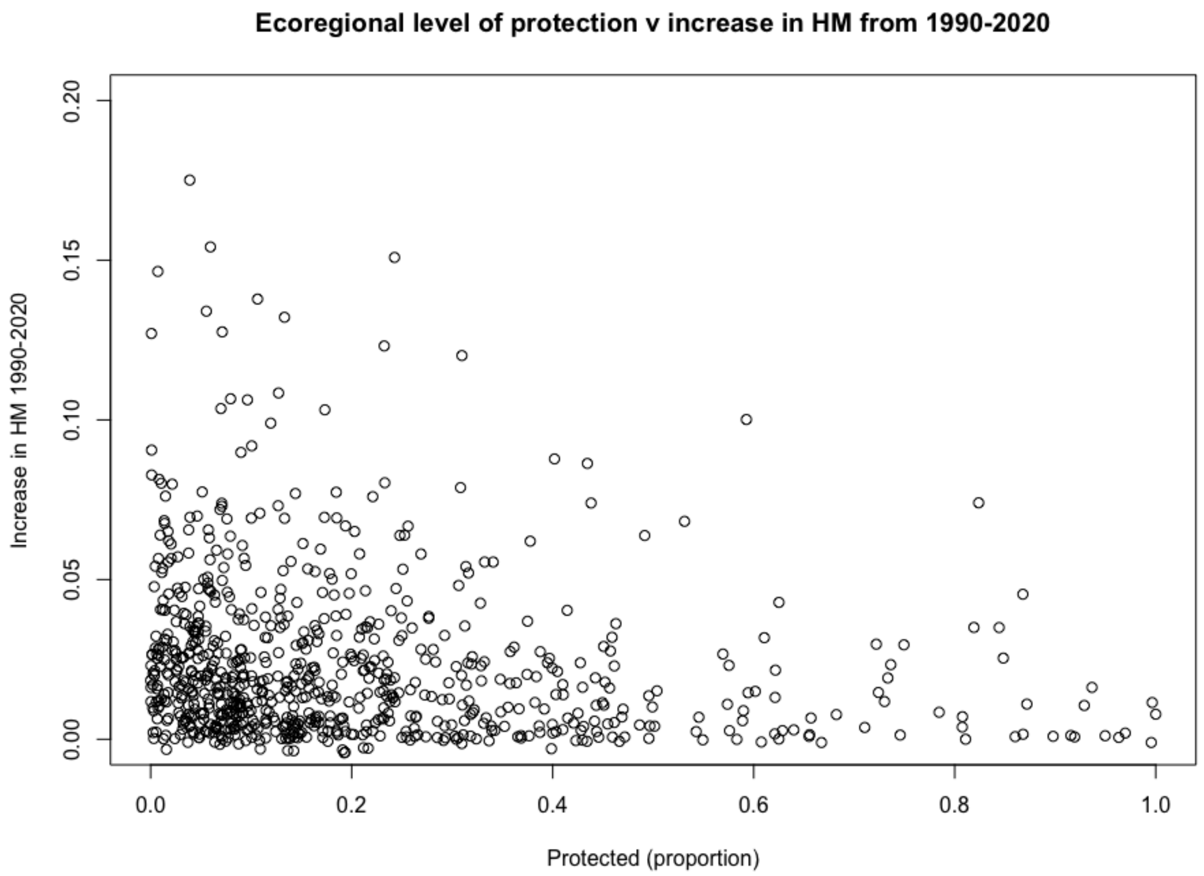

Supplementary Figure 15. A bivariate map showing the percentage protected in 2024 vs. the change in human modification (HM_{2020} - HM_{1990}).

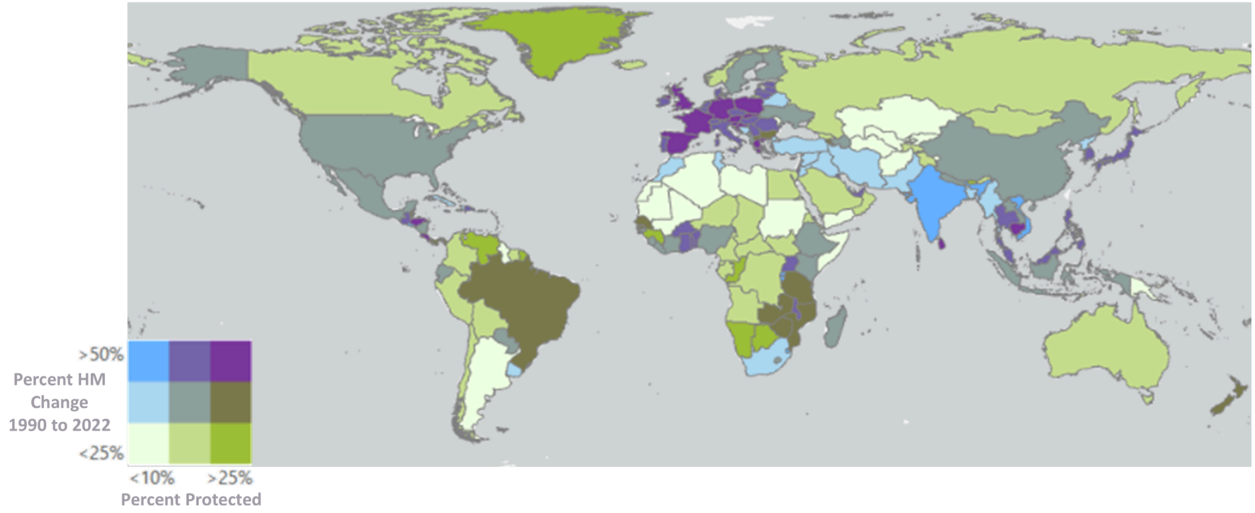

Supplementary Table 1. Derivation of human modification intensity values (I_s) by land use classification from the landscape development intensity index (LDI), calculated as max-normalized empower densities[30].

Land use	LDI	I_s
Natural system	1	0.000
Natural open water	1	0.000
Pine plantation	1.58	0.064
Rec/open space low-intensity	1.83	0.092
Woodland pasture (w/livestock)	2.02	0.113
Improved past (w/o livestock)	2.77	0.197
Improved past - low intensity (w/livestock)	3.41	0.268
Citrus plantation	3.68	0.298
Improved pasture high-intensity (w/livestock)	3.74	0.304
Row crops	4.54	0.393
Single family low density	6.9	0.656
Rec/open space high-intensity	6.92	0.658
Ag high intensity	7	0.667
Single family medium density	7.57	0.730
Single family high density	7.55	0.728
Mobile home medium density	7.7	0.744
Highway 2 lane	7.81	0.757
Low intensity commercial	8	0.778
Institutional	8.07	0.786
Highway 4 lane	8.28	0.809
Mobile home high density	8.29	0.810
Industrial	8.32	0.813
Multi-family res low-rise	8.66	0.851
High intensity commercial	9.18	0.909
Multi-family res high-rise	9.19	0.910
Commercial/business district, 2 stories	9.42	0.936
Commercial/business district, 4 stories	10	1.000

Supplementary Table 2. ESA CCI Land cover classes examined ("class name") and used to define pattern of grazing patches, andused to estimate grazing footprint from the livestock density for bovines, goats, and sheep (LU/km2) from the Gridded Livestock of the World[51] (GLW). LU= livestock units. Grazing classes were first identified by identifying cover classes typically associated with grazing from a biological perspective (column "biological"). Adjusted classes (column "adjusted") reflect subsequent evaluation based on the empirical distribution of livestock grazing for each class, and the percent of land in each class, as well as visual inspection of the spatial overlapping pattern of the grazing density against the land cover map.

		**Livestock units/km2 value at percentile**			**Grazing classes**		
**Value**	**Class name**	**50%**	**90%**	**95%**	**Biological**	**Adjusted**	**% of weighted area**
0	No data						
10	Cropland, rainfed	27.9	156.0	220.0			11.765%
11	Cropland, rainfed herbaceous	11.7	78.1	110.3			9.662%
12	Cropland, rainfed tree or shrub	23.0	103.8	168.3			0.242%
20	Cropland, irrigated	92.0	340.0	444.0			4.438%
30	Mosaic cropland (>50%)	23.5	103.4	135.6	Y	Y	4.801%
40	Mosaic cropland (<50%)	23.3	87.2	135.7	Y	Y	4.238%
50	Tree (>15%), broadleaved, evergreen	2.1	27.7	51.8			6.860%
60	Tree (>15%), broadleaved, deciduous	5.9	44.4	44.4		Y	4.277%
61	Tree (>40%), broadleaved, deciduous	11.9	35.7	51.6			0.678%
62	Tree (>15-40%), broadleaved, deciduous	11.8	59.8	99.9		Y	3.628%
70	Tree (>15%), needleleaved, evergreen	1.0	17.9	34.0			2.607%
71	Tree (>40%), needleleaved, evergreen	0.3	0.3	0.3			0.086%
72	Tree (>15-40%), needleleaved, evergreen	0.2	0.3	1.4			0.000%
80	Tree (>15%, needleleaved, deciduous	0.8	0.8	0.8			0.412%
81	Tree (>40%), needle leaved, deciduous	51.2	183.1	249.3		Y	0.006%
82	Tree (>15-40%), needleleaved, deciduous	127.0	127.0	127.0		Y	0.000%
90	Tree, mixed leaf type	3.0	3.0	3.0			0.724%
100	Mosaic tree/shrub (>50%)	7.7	45.4	77.6	Y	Y	2.805%
110	Mosaic herbaceous (>50%)	11.6	67.9	107.7	Y	Y	1.011%
120	Shrubland	8.5	44.6	77.5	Y	Y	10.135%

121	Shrubland, evergreen	9.9	34.0	54.0	Y	Y	0.179%
122	Shrubland, deciduous	2.2	35.9	67.7	Y	Y	1.147%
130	Grassland	5.4	71.3	103.6	Y	Y	13.217%
140	Lichens and mosses	0.3	0.3	0.3			0.094%
150	Sparse vegetation (<15%)	2.4	23.0	55.3	Y	Y	4.303%
151	Sparse tree (<15%)	17.1	23.8	23.8			0.000%
152	Sparse shrub (<15%)	41.9	106.0	122.1	Y	Y	0.066%
153	Sparse herbaceous (<15%)	42.0	110.0	126.2	Y	Y	0.469%
160	Tree, flooded	1.0	1.0	1.0			0.238%
170	Tree, flooded saline	2.4	52.0	83.3			0.110%
180	Shrub/herbaceous flooded	6.1	43.6	77.4	Y	Y	0.923%
190	Urban areas	5.9	103.7	167.2			0.859%
200	Bare	2.4	35.7	51.8			8.638%
201	Bare areas	0.6	74.0	122.0			NA
202	Bare areas	5.4	21.6	41.4			NA
210	Water	1.3	35.8	76.0			1.138%
220	Permanent snow & ice	0.3	4.9	6.9			0.107%

Supplementary Table 3. The area and percentage of lands dominated by threat classes.

Threat class	Percentage	Area (M km2)
HM<0.01	46.98%	61.74
Agriculture	39.60%	52.05
Residential & commercial devel.	1.59%	2.10
Energy production & mining	0.22%	0.29
Biological resource use	1.00%	1.32
Human accessibility	5.10%	6.70
Natural process modification	0.04%	0.05
Pollution	0.59%	0.77
Transportation & service corridors	4.87%	6.40

Supplementary Table 4. Time-series of mean human modification values (HMv3 300 m) from 1990 to 2020 for the four example areas: Asuncion, Paraguay; Calgary, Canada; Qing Yuan, China; and Timbuktu, Mali.

HM v3 300 m	Asuncion, Paraguay	Calgary, Canada	Qing Muay, China	Timbuktu, Mali
**1990**	0.1081	0.2765	0.2416	0.0470
**1995**	0.1129	0.2800	0.2599	0.0472
**2000**	0.1157	0.2834	0.2812	0.0480
**2005**	0.1163	0.2852	0.3011	0.0489
**2010**	0.1220	0.2890	0.3252	0.0531
**2015**	0.1257	0.2927	0.3422	0.0543
**2020**	0.1289	0.2947	0.3482	0.0571

Supplementary Table 5. Overview comparison of human modification to other human pressure methods.

Name	Human modification v3 (2022)	Human modification 2017 (HM2017)	Human modification 2016 (HM2016)	Low impact areas (LIA)	Human footprint (HFP-100)	Human footprint (HF 2000-2013)	Human footprint (HF 1993-2009)	Human Influence Index (HII)
Citation	(this paper)	Theobald et al. (2020)	Kennedy et al. (2019); Theobald (2013)	Jacobsen et al. (2019)	Gassert et al. (2023)	Williams et al. (2020)	Venter et al. (2016)	Sanderson et al. (2002)
Conceptual framework	IUCN Direct Threats Classification v2 (Salafsky et al., 2008); Theobald et al. (2020)	Direct Threats Classification v2 (Salafsky et al., 2008)	Direct Threats Classification v2 (Salafsky et al., 2008)	Classed overlays (as primarily modified or used by humans)	Based on Sanderson et al. (2002) framework, a "simpler" approach than HM	Based on Venter et al. (2016).	Based on Sanderson et al. (2002) framework	Ad hoc
Footprint/intensity	Intensity values based on land development index (LDI; Brown and Vivas, 2005); Kennedy et al. 2019 SI; McBride et al. 2012 elicitation procedure	Intensity values based on land development index (LDI; Brown and Vivas, 2005); Kennedy et al. 2019 SI; McBride et al. 2012 elicitation procedure	Intensity values based on land development index (LDI; Brown and Vivas, 2005); Kennedy et al. 2019 SI; McBride et al. 2012 elicitation procedure	-	Authors estimated 0-10 index for each pressure.	Authors estimated 0-10 index for each pressure	Authors estimated 0-10 index for each pressure	Authors estimated 0-10 index for each pressure
Resolution	90, 300 m	0.3 km	1 km	1 km	100 m	1 km	1 km	1 km
Year(s)	~2022<br>1990-2020 (5 years)	~2017<br>1990-2015	~2016	~2016	2015-2019, 2020 (v1.2 2017-2021)	2013<br>2000-2013	2009<br>1993-2009	~2000
Threat classes								

**Agriculture**	ESA LCCS land cover (300 m) ESA LCCS land cover (100 m) Global cropland database (30 m) Plantations (30 m) Grazing (GLW v2; 1km)	Cropland & pastureland for 1990, 2015 (ESA CCI; 300 m) and cropland intensity (GLS; 1 km); Unified Cropland Layer (UCL; 1 km); grazing (GLW; 10 km; 1	Unified Cropland Layer (UCL; 1 km); grazing (GLW v2; 1 km; LU km-2	ESA LCCS land cover: urban & cropland Grazing Gridded livestock World and LandScan Population (>1->16 people/km2)	Cropland from Copernicus CGLS-LC100 (for 2015-2019) and ESRI 2020 Global Land UseLand Cover (for 2020) Pastureland from Ramankutty et al. 2008 (10 km resolution* not 1 km downsampled as stated).	Cropland (ESA CCI land cover 300 m for 2000 and 2013) Pastureland for 2000 from Ramankutty et al. (2008), 10 km resolution, downsampled to 1 km)	Cropland (University of Maryland for 1990 and GlobCover for 2009); pastureland (2000), 10 km	Land cover 1993
**Residential & commercial development**	Global Human Settlement Layer vR2022A (GHSL; 100 m); GHSL 10 m; Microsoft Building Footprints Impervious surface (10 m) Land cover (10 m)	GHSL vR2019 300 m	GHSL v2016 1 km; Population density (GPW v4 2015; 1 km)	Nighttime lights (VIIRS > 0)	Built-up class from Copernicus CGLS-LC100 (for 2015-2019) and ESRI 2020 Global Land UseLand Cover (for 2020) Human population density from WorldPop 100 m resolution (not 100 m2 as erroneously stated)	Nighttime lights (DMSP OLS >20; 1 km; 1994–2012); Population density (CIESIN v3; 4 km; 1990, 2010)	Nighttime lights (DMSP OLS >20; 1 km; 1994–2012); population density (CIESIN v3; 4 km; 1990, 2010)	Gridded Population of the World (1995) Land cover 1993 Nightlights (DMSP/OLS; 1994)
**Energy production & mining**	Oil & gas production (gas flares; DMSP OLS and VIIRS); renewable and nonrenewable power plants	Oil & gas production (gas flares; DMSP OLS and VIIRS); renewable and nonrenewable power plants	Oil & gas wells, wind turbines, mines (OSM, 2016)	-		-	-	-

	(WRI); large mining operations (Maus)	(WRI); large mining operations (S&P)							
**Biological resource use**	Forest loss (Hansen et al., 2013; 0.03–1 km; 2000–2023)	Forest loss (Hansen et al., 2013; Curtis et al., 2018; 0.03–1 km; 2000–2017)	-	Forest loss (Hansen et al., 2013); Burned area extent (MODIS)	-	-	-	-	-
**Human accessibility (intrustions)**	Human intrusion (HUE, 1990–2020; 1 km) - accessibility along transportation network and navigable rivers/coastlines, weighted by settlement population	Human intrusion (HUE, 1990–2015; 1 km) - accessibility along transportation network and navigable rivers/coastlines, weighted by settlement population	-	-	Navigable waterways (using VMAP)	Navigable waterways (using VMAP)	Navigable waterways (using VMAP)	Navigable waterways (using VMAP)	
**Natural system mod.**	Reservoirs (GRanD, 1990–2017; 0.03 km; GeoDAR 30 m; RealSAT 30 m)	Reservoirs (GRanD, 1990–2017; 0.03 km)	-	-	-	-	-	-	
**Pollution**	Nitrous oxide pollution (Sentinel 5P 0.5 km; EDGAR, 1990–2012; 100 km)	Nitrous oxide pollution (EDGAR, 1990–2012; 100 km)	-	-	-	-	-	-	

**Transportation**	Road (highway, minor, two-track; OSM, 2024); railways (OSM, 2024) land use (OSM, 2024); power lines (OSM, 2024); electrical power infrastructure (harmonized DMSP and VIIRS, 1992–2023)	Road (highway, minor, two-track; OSM, 2019); railways (OSM, 2019) power lines (OSM, 2019); electrical power infrastructure (harmonized DMSP and VIIRS, 1992–2018)	Road (highway, minor, two-track; OSM, 2016; gROADS-2000); railways (OSM, 2016; VMAP-2000), power lines (OSM, 2016); electric infrastructure (nighttime lights; DMSP OLS, 2013)	-	Roads and railways (no differentiation of type) from OSM (2021 source). Mapped as 80% with 0.5 buffer VIIRS nightime lights (using 25 as maximum)	Roads (gROADS, 1980–2000) and Open Streetmap (OSM) railways (VMAP0-2000). Mapped as 40% 1 to 15 km decay.	Roads (gROADS, 1980–2000) railways (VMAP0-2000). Mapped as 40% 1 to 15 km decay.	Roads (VMAP 1990s)
**Analyses**								
**Integration formula**	Increasive to 1.0 using fuzzy sum (averaging multiple datasets into individual threats)	Increasive to 1.0 using fuzzy sum (averaging multiple datasets in a threat)	Increasive to 1.0 using fuzzy sum	Overlayed excluded areas (added WDPA Cat I-IV)	Summed (additive)	Summed (additive)	Summed (additive)	Summed (additive)
**Metric**	H; 0–1.0 continuous value	H; 0–1.0 continuous value	H; 0–1.0 continuous value	0/1	Ordinal value	Ordinal value	Ordinal value	-
**"Wild"**	H<0.084 (weighted mean H in WDPA Cat I-II)	H<0.084 (mean H in WDPA Cat I-II)	H<0.084 (mean H in WDPA Cat I-II)	0/1	HF < 4	HF < 4	HF < 4	10% cutoff (HF 3-18)
**Uncertainty analysis**	Calculates per-pixel variance due to estimates of intensity values, randomized (n=100). Calculate	Calculates per-pixel variance due to estimates of intensity values, randomized (n=50)	Calculates per-pixel variance due to estimates of intensity values, randomized (n=100)	-	-	-	-	-

	uncertainty associated with primary land user/cover datasets.							
**Validation**	Tested using independent validation dataset that included ~10 000 subplots within ~1000 0.36 km2 sample plots stratified by urban, exurban, and rural areas.	Tested using independent validation dataset that included ~10 000 subplots within ~1000 0.36 km2 sample plots	Tested using independent validation dataset that included ~10 000 subplots within ~1000 0.36 km2 sample plots	Tested using independent validation, random distribution of 3,460 1 km2 sample plots	Tested using independent validation, random distribution of 3,460 1 km2 sample plots	Tested using independent validation, random distribution of 3,460 1 km2 sample plots	Tested using independent validation, random distribution of 3,460 1 km2 sample plots	-
**Assumption of edge-effects**	Not modeled directly, and so provides foundation for subsequent fragmentation and connectivity analyses	Not modeled directly, and so provides foundation for subsequent fragmentation and connectivity analyses	Not modeled directly, and so provides foundation for subsequent fragmentation and connectivity analyses	-	Buffer 15 km from roads (declining)	Buffer 12 km from roads (declining); 10 km smoothing on cropland/pasture ?	Buffer 12 km from roads (declining); 10 km smoothing on cropland/pasture ?	Buffer 12 km (declining)

Citations for Supplementary Table 5

Brown, M.T. and Vivas, M.B., 2005. Landscape development intensity index. *Environmental Monitoring and Assessment*, 101, pp.289-309.

Du, Z., Yu, L., Yang, J., Xu, Y., Chen, B., Peng, S., Zhang, T., Fu, H., Harris, N. and Gong, P., 2022. A global map of planting years of plantations. *Scientific Data*, 9(1), pp.1-9.

Elvidge, C., Ziskin, D., Baugh, K., Tuttle, B., Ghosh, T., Pack, D., Erwin, E., and Zhizhin, M.: A Fifteen Year Record of Global Natural Gas Flaring Derived from Satellite Data, *Energies*, 2, 595, https://doi.org/10.3390/en2030r0595, 2009.

Elvidge, C., Zhizhin, M., Baugh, K., Hsu, F. C., and Ghosh, T.: Methods for global survey of natural gas flaring from visible infrared imaging radiometer suite data, *Energies*, 9, 14, https://doi.org/10.3390/en9010014, 2016.

Elvidge, C. D., Imhoff, M. L., and Baugh, K. E.: Night-time lights of the world: 1994–1995, ISPRS J. *Photogramm. Remote S.*, 56, 81–99, 2001.

Elvidge, C. D., Baugh, K. E., Zhizhin, M., and Hsu, F. C.: Why VIIRS data are superior to DMSP for mapping nighttime lights, *Proceedings of the Asia-Pacific Advanced Network*, 35, 62–69, 2013.

European Space Agency (ESA). 2017. *Land Cover CCI Product User Guide version 2.0.* Document ref: CCI-LC-PUGv2.

Gassert, F., Venter, O., Watson, J.E., Brumby, S.P., Mazzariello, J.C., Atkinson, S.C. and Hyde, S., 2023. An operational approach to near real time global high resolution mapping of the terrestrial Human Footprint. *Frontiers in Remote Sensing*, 4, p.1130896.

Geldmann, J., Barnes, M., Coad, L., Craigie, I.D., Hockings, M. and Burgess, N.D., 2013. Effectiveness of terrestrial protected areas in reducing habitat loss and population declines. *Biological Conservation*, 161, pp.230-238.

Ghosh, T., Baugh, K.E., Elvidge, C.D., Zhizhin, M., Poyda, A. and Hsu, F.C., 2021. Extending the DMSP nighttime lights time series beyond 2013. Remote Sensing, 13(24), p.5004.

Gilbert, Marius; Cinardi, Giuseppina; Da Re, Daniele; Wint, William G. R.; Wisser, Dominik; Robinson, Timothy Pt., 2022, "Global buffaloes distribution in 2015 (5 minutes of arc)", https://doi.org/10.7910/DVN/I1WCAB, Harvard Dataverse, V1

Gilbert, Marius; Cinardi, Giuseppina; Da Re, Daniele; Wint, William G. R.; Wisser, Dominik; Robinson, Timothy P., 2022, "Global cattle distribution in 2015 (5 minutes of arc)", https://doi.org/10.7910/DVN/LHBICE, Harvard Dataverse, V1

Gilbert, Marius; Cinardi, Giuseppina; Da Re, Daniele; Wint, William G. R.; Wisser, Dominik; Robinson, Timothy P., 2022, "Global goats distribution in 2015 (5 minutes of arc)", https://doi.org/10.7910/DVN/YYG6ET, Harvard Dataverse, V1

Gilbert, Marius; Cinardi, Giuseppina; Da Re, Daniele; Wint, William G. R.; Wisser, Dominik; Robinson, Timothy P., 2022, "Global horses distribution in 2015 (5 minutes of arc)", https://doi.org/10.7910/DVN/JJGCTX, Harvard Dataverse, V1

Gilbert, Marius; Cinardi, Giuseppina; Da Re, Daniele; Wint, William G. R.; Wisser, Dominik; Robinson, Timothy P., 2022, "Global sheep distribution in 2015 (5 minutes of arc)", https://doi.org/10.7910/DVN/VZOYHM, Harvard Dataverse, V1

Global Energy Observatory, Google, KTH Royal Institute of Technology in Stockholm, Enipedia, World Resources Institute. 2021. Global Power Plant Database version 1.3.0. Accessed through https://datasets.wri.org/datasets/global-power-plant-database on March 23, 2021.

Hansen, M. C., Potapov, P. V., Moore, R., Hancher, M., Turubanova, S. A. A., Tyukavina, A., and Kommareddy, A. 2013. High-resolution global maps of 21st-century forest cover change, *Science*, 342, 850–853.

Huang, X., Li, J., Yang, J. et al. 30 m global impervious surface area dynamics and urban expansion pattern observed by Landsat satellites: From 1972 to 2019. *Sci. China Earth Sci.* (2021). https://doi.org/10.1007/s11430-020-9797-9.

IUCN 2022. IUCN Threats Classification Scheme (v3.3; URL, visited 20230615).

Kennedy, C.M., Oakleaf, J.R., Theobald, D.M., Baruch-Mordo, S. and Kiesecker, J., 2019a. Managing the middle: A shift in conservation priorities based on the global human modification gradient. *Global Change Biology*, 25(3), pp.811-826.

Kennedy, C.M., Oakleaf, J.R., Theobald, D.M., Baruch-Mordo, S. and Kiesecker, J., 2019b. Supplementary material to: Managing the middle: A shift in conservation priorities based on the global human modification gradient. https://onlinelibrary.wiley.com/action/downloadSupplement?doi=10.1111%2Fgcb.14549&file=gcb14549-sup-0001-AppendixS1.pdf.

Lamarche, C., Santoro, M., Bontemps, S., d'Andrimont, R., Radoux, J., Giustarini, L., Brockmann, C., Wevers, J., Defourny, P. and Arino, O., 2017. Compilation and validation of SAR and optical data products for a complete and global map of inland/ocean water tailored to the climate modeling community. *Remote Sensing*, 9(1), p.36.

Lehner, B., Liermann, C.R., Revenga, C., Vörösmarty, C., Fekete, B., Crouzet, P., Döll, P., Endejan, M., Frenken, K., Magome, J. and Nilsson, C., 2011. High-resolution mapping of the world's reservoirs and dams for sustainable river-flow management. *Frontiers in Ecology and the Environment*, 9(9), pp.494-502.

Maus, V., Giljum, S., da Silva, D.M., Gutschlhofer, J., da Rosa, R.P., Luckeneder, S., Gass, S.L., Lieber, M. and McCallum, I., 2022. An update on global mining land use. *Scientific Data*, 9(1), pp.1-11.

Mazur, E., M. Sims, E. Goldman, M. Schneider, M. Daldoss Pirri, C.R. Beatty, F. Stolle, and M. Stevenson. 2024. *SBTN Natural Lands Map - Technical Documenation, v1.0*. Unpublished report, World Resources Institute, Systemiq, and World Wildlife Fund - United States. [URL: accessed 16 September 2024].

Mu, H., Li, X., Wen, Y., Huang, J., Du, P., Su, W., Miao, S. and Geng, M., 2022. A global record of annual terrestrial Human Footprint dataset from 2000 to 2018. *Scientific Data*, 9(1), p.176

NASA 2023. MODIS burned area MCD64A1.061.

Nechaev, D., Zhizhin, M., Poyda, A., Ghosh, T., Hsu, F.C. and Elvidge, C., 2021. Cross-sensor nighttime lights image calibration for DMSP/OLS and SNPP/VIIRS with residual U-net. *Remote Sensing*, 13(24), p.5026.

Omara, M., Gautam, R., O'Brien, M., Himmelberger, A., Franco, A., Meisenhelder, K., Hauser, G., Lyon, D., Chulakadaba, A., Miller, C. and Franklin, J., 2023. Developing a spatially explicit global oil and gas infrastructure database for characterizing methane emission sources at high resolution. *Earth System Science Data Discussions*, 2023, pp.1-35.

OpenStreetMap 2024. Data retrieved July 16, 2024. https://www.openstreetmap.org.

Pesaresi, M., Ehrilch, D., Florczyk, A. J., Freire, S., Julea, A., Kemper, T., Soille, P., and Syrris, V. *GHS built-up grid, derived from Landsat, multitemporal (1975, 1990, 2000, 2015)*, European Commission, Joint Research Centre, JRC Data Catalogue, Aspra, Italy, 2015.

Phillpott, M., O'Connor, J., Ferreira, A., Max, S., Kruitwagen, L., & Guzzardi, M. (2024). Solar Asset Mapper: A continuously-updated global inventory of solar energy facilities built with satellite data and machine learning (1.0) [Data set]. Zenodo. https://doi.org/10.5281/zenodo.11368204.

Potapov, P., Hansen, M.C., Pickens, A., Hernandez-Serna, A., Tyukavina, A., Turubanova, S., Zalles, V., Li, X., Khan, A., Stolle, F. and Harris, N., 2022b. The global 2000–2020 land cover and land use change dataset derived from the Landsat archive: first results. *Front. Remote Sens*, 3.

Potapov, P., Turubanova, S., Hansen, M.C., Tyukavina, A., Zalles, V., Khan, A., Song, X.P., Pickens, A., Shen, Q. and Cortez, J., 2022a. Global maps of cropland extent and change show accelerated cropland expansion in the twenty-first century. *Nature Food*, 3(1), pp.19-28.

Ramankutty, N., Evan, A.T., Monfreda, C. and Foley, J.A., 2008. Farming the planet: 1. Geographic distribution of global agricultural lands in the year 2000. *Global Biogeochemical Cycles*, 22(1).

Robinson, T. P., Wint, G. W., Conchedda, G., Van Boeckel, T. P., Ercoli, V., Palamara, E., and Gilbert, M.: Mapping the global distribution of livestock, PloS one, 9, e96084, https://doi.org/10.1371/journal.pone.0096084, 2014.

Salafsky, N., Salzer, D., Stattersfield, A.J., Hilton-Taylor, C., Neugarten, R., Butchart, S.H., Collen, B.E.N., Cox, N., Master, L.L., O'Connor, S. and Wilkie, D., 2008. A standard lexicon for biodiversity conservation: unified classifications of threats and actions. *Conservation Biology*, 22(4du), pp.897-911.

Sanderson, E.W., Jaiteh, M., Levy, M.A., Redford, K.H., Wannebo, A.V. and Woolmer, G., 2002. The human footprint and the last of the wild: the human footprint is a global map of human influence on the land surface, which suggests that human beings are stewards of nature, whether we like it or not. *BioScience*, 52(10), pp.891-904.

Schiavina M., Freire S., MacManus K. (2022): GHS-POP R2022A - GHS population grid multitemporal (1975-2030). European Commission, Joint Research Centre (JRC) doi:10.2905/D6D86A90-4351-4508-99C1-CB074B022C4A.

Tang, L. and Werner, T.T., 2023. Global mining footprint mapped from high-resolution satellite imagery. *Communications Earth & Environment*, 4(1), p.134.

Thenkabail, P.S., Teluguntla, P.G., Xiong, J., Oliphant, A., Congalton, R.G., Ozdogan, M., Gumma, M.K., Tilton, J.C., Giri, C., Milesi, C., Phalke, A., Massey, R., Yadav, K., Sankey, T., Zhong, Y., Aneece, I., and Foley, D., 2021, *Global Cropland-Extent Product at 30-m Resolution (GCEP30) Derived from Landsat Satellite Time-Series Data for the Year 2015 Using Multiple Machine-Learning Algorithms on Google Earth Engine Cloud*: U.S. Geological Survey Professional Paper 1868, 63 p., https://doi.org/10.3133/pp1868.

Theobald, D.M., Kennedy, C., Chen, B., Oakleaf, J., Baruch-Mordo, S. and Kiesecker, J., 2020. Earth transformed: detailed mapping of global human modification from 1990 to 2017. *Earth System Science Data*, 12(3), pp.1953-1972.

TROPOMI 2023. Copernicus Sentinel-5P mission performance centre nitrogen dioxide readme. Report S5P-MPC-KNMI-PRF-NO2. URL: https://sentinel.esa.int/documents/247904/3541451/Sentinel-5P-Nitrogen-Dioxide-Level-2-Product-Readme-File (accessed 22 March 2023).

Venter, O., Sanderson, E. W., Magrach, A., Allan, J. R., Beher, J., Jones, K. R., Possingham, H. P., Laurance, W. F., Wood, P., Fekete, B. M., Levy, M. A., Watson, J. E. M.: Global terrestrial Human Footprint maps for 1993 and 2009, *Sci. Data*, 3, 160067, https://doi.org/10.1038/sdata.2016.67, 2016.

Wang, J., Walter, B.A., Yao, F., Song, C., Ding, M., Maroof, A.S., Zhu, J., Fan, C., Xin, A., McAlister, J.M. and Sikder, S., 2021. GeoDAR: Georeferenced global dam and reservoir dataset for bridging attributes and geolocations. *Earth System Science Data Discussions*, 2021, pp.1-52.

Zanaga, D., Van De Kerchove, R., Daems, D., De Keersmaecker, W., Brockmann, C., Kirches, G., Wevers, J., Cartus, O., Santoro, M., Fritz, S. and Lesiv, M., 2022. *ESA WorldCover 10 m 2021 v200*.

Zhang, M., Wu, B., Zeng, H., He, G., Liu, C., Tao, S., Zhang, Q., Nabil, M., Tian, F., Bofana, J. and Beyene, A.N., 2021. GCI30: a global dataset of 30 m cropping intensity using multisource remote sensing imagery. *Earth System Science Data*, 13(10), pp.4799-4817.

Zhao, C., Cao, X., Chen, X., & Cui, X.. (2020). A Consistent and Corrected Nighttime Light dataset (CCNL 1992-2013) from DMSP-OLS data. https://doi.org/10.5281/zenodo.6644980.

Williams, B.A., Venter, O., Allan, J.R., Atkinson, S.C., Rehbein, J.A., Ward, M., Di Marco, M., Grantham, H.S., Ervin, J., Goetz, S.J. and Hansen, A.J., 2020. Change in terrestrial human footprint drives continued loss of intact ecosystems. *One Earth*, 3(3), pp.371-382.
